# Use of Halogenated Units for the Construction of Artificial Carbohydrate Receptors

**DOI:** 10.3390/molecules31081237

**Published:** 2026-04-09

**Authors:** Betty Fuhrmann, Conrad Hübler, Monika Mazik

**Affiliations:** Institut für Organische Chemie, Technische Universität Bergakademie Freiberg, Leipziger Straße 29, 09599 Freiberg, Germany

**Keywords:** molecular recognition, halogenated building blocks, carbohydrate receptors, intramolecular interactions, NMR titrations, water-containing solvent, hydrogen bonds, halogen bonds, supramolecular chemistry, steric effects

## Abstract

To investigate the potential of halogen-containing building blocks in the development of artificial carbohydrate receptors, the 1,3,5-trisubstituted 2,4,6-triethylbenzene scaffold with halogenated subunits and classical hydrogen bonding sites was used as a model system. In the first studies, the influence of the presence of halogens on the binding properties of compounds bearing benzamidomethyl units was investigated, whereby the type of halogen and its ring position were varied. The question was whether the presence of halogens could lead to an increase in binding effectivity and whether this increase can be attributed to the formation of halogen bonds (especially for X = Br and I in *ortho* position) with the sugar substrate or to other effects. The binding studies revealed some interesting relationships between structure and binding affinity for the tested compounds **1**–**9**. For those bearing the halogen substituent in the *ortho* position to the amide functionality, the binding affinity increases in the expected order **4** (*o*-F) < **3** (*o*-Cl) < **2** (*o*-Br) < **1** (*o*-I). In the presence of small amounts of water in CDCl_3_, an increase in binding strength was observed in comparison to experiments conducted in dry CDCl_3_. The present studies aim to provide impulses for the use of halogenated building blocks in the design of artificial carbohydrate receptors. Optimizing the type of halogenated units and the receptor architecture should result in more effective carbohydrate receptors capable of functioning effectively in aqueous media through a combination of different noncovalent interactions.

## 1. Introduction

**Figure 1:**
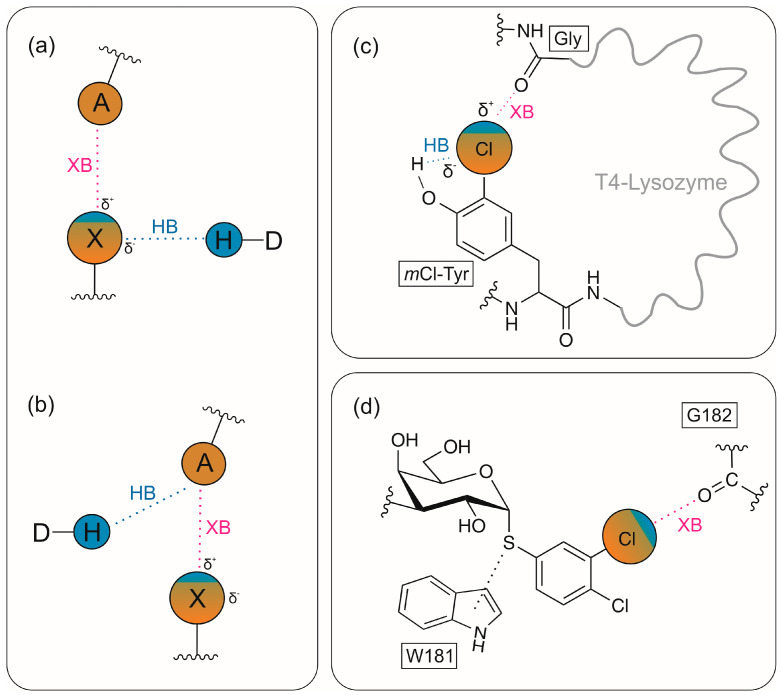
(**a**) Schematic illustration of HBeXB (hydrogen bond-enhanced halogen bond): DH⋯X hydrogen bond (HB) to the electron-rich region of a halogen X, which simultaneously acts as a halogen bond donor (XB) for an acceptor A, and (**b**) simultaneously occurring DH⋯A hydrogen bonding (HB) and X⋯A halogen bond (XB) (adapted from ref. [25]). (**c**) Schematic illustration of HBeXB in the modified T4 lysozyme, in which the tyrosine moiety in position 18 (Y18) has been replaced by *m*-chlorotyrosine (*m*ClY) [27]. (**d**) Cl⋯O=C halogen bond (XB) and sulfur⋯π interaction in the complex of galectin-3 with a carbohydrate-based inhibitor designed by Nilsson, Zetterberg et al. [28].

Carbohydrate-mediated biological recognition processes are characterized by the occurrence of multiple noncovalent interactions, in particular, neutral and charge-enhanced hydrogen bonds, CH⋯π interactions and van der Waals forces. The binding mode used by carbohydrate-binding proteins, such as lectins, has mainly been determined on the basis of the crystal structures of their complexes with different carbohydrates [1,2,3,4,5,6,7,8,9]. The way in which carbohydrate substrates are bound by proteins has inspired the design of molecules with the ability to act as artificial carbohydrate-binding agents (artificial carbohydrate receptors; for examples of review articles, see refs. [10,11,12,13,14,15,16,17,18,19,20,21,22,23,24]). The consideration of additional noncovalent interactions in the design of artificial receptors, such as halogen bonds (see Figure 1a,b), represents a promising research approach in the development of effective and selective receptor molecules. In this context, synergistic effects between hydrogen (HB) and halogen bonds (XB) can play an important role. Particularly noteworthy is the synergistic relationship, in which the halogen bond is reinforced by a hydrogen bond [25,26] (HBeXB = hydrogen bond-enhanced halogen bonds), as schematically shown in Figure 1a. The biological relevance of such synergistic interactions can be seen, for example, in the development of enzymes whose stability and activity could be increased by HBeXB (Figure 1c). By replacing a tyrosine residue in T4 lysozyme with the unnatural amino acid *m*-chlorotyrosine, an increase in both thermal stability and enzymatic activity at elevated temperatures was achieved [27]. The XB donor potential of Cl is, in this case, enhanced by polarization through intramolecular HB from the adjacent OH group.

In the design of artificial carbohydrate receptors, the selection of suitable halogen-containing building blocks and their incorporation into a scaffold in a way that ensures effective intermolecular interactions with the binding partner represents the key challenge.

At this point, it should also be noted that in developing glycomimetic ligands, which play an important role in carbohydrate-based drug discovery, for example, the use of halogenated substituents has already been considered [28,29,30], such as in the case of the galactose-based lectin inhibitors bearing a halogeno-substituted phenyl group [28]. The structure–activity relationship studies revealed that the presence of the additional 4-chloro group in the benzene ring (Figure 1d) led to an approximately 2-fold increase in affinity towards galectin-3 compared to the monosubstituted 3-chloro analogue (increase in halogen bonding strength due to the introduction of an electron-withdrawing group in the vicinity).

As often discussed in the literature halogen and hydrogen bonds have some similarities in terms of geometry and energy, but they also have important differences (for examples of review articles, see refs. [31,32,33,34]). For example, halogen bonds have a stronger preference for linearity than hydrogen bonds, are more hydrophobic in nature and exhibit different dependencies on solvents. Above all, the more pronounced hydrophobic character can play a very positive role for effective substrate binding in aqueous media (for a review on molecular recognition processes in water, see ref. [35]).

The aim of this work was to investigate the binding properties of model systems containing both halogenated building blocks and classical hydrogen bond donor/acceptor sites, as described in Section 2.1.

## 2. Results and Discussions

### 2.1. Selection of the Model System

**Figure 2:**
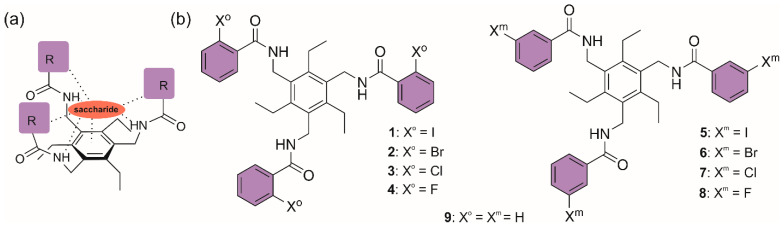
(**a**) Schematic representation of the receptor–sugar complex. (**b**) Structures of the target compounds with halogen substituents in *ortho* (**1**–**4**) and *meta* (**5**–**8**) positions to the CONH functionalities and without halogen substituents (**9**) (reference compound).

For the initial studies, we have decided to investigate the influence of the presence of halogens on the binding properties of triethylbenzene derivatives **1**–**8** bearing three benz-amidomethyl units [(benzoylamino)methyl units] (Figure 2). The type of the halogen and its ring position (*ortho* or *meta*) were varied. According to earlier studies, the halogen-free compound **9** [36] binds only weakly to glycosides in organic media such as chloroform. The question was whether the presence of halogens could lead to an increase in binding effectivity and whether this increase can be attributed to the formation of halogen bonds (especially for X = Br and I in *ortho* position) with the sugar substrate or to other effects.

**Figure 3:**
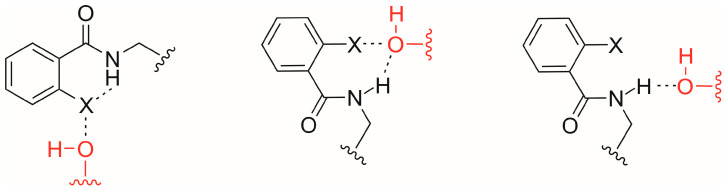
Examples of possible binding motifs in the receptor–glucoside complexes involving the compounds with halogen substituent in *ortho* position (**1**–**4**) to the CONH functionality.

On the one hand, the presence of the halogen influences the hydrogen bond (HB) donor capacity of NH; on the other hand, the presence of the CONH group in the *ortho* position favours the halogen bond (XB) donor effectiveness of X (in contrast to the *meta*-substituted analogues). Despite the simple structure of the compounds described here, it is necessary to take into account a variety of factors that have an influence on the binding properties. These factors also include the possible formation of intramolecular NH⋯X hydrogen bonds and their influence on the bonding properties (particularly in the case of X = F). The competition between inter- and intramolecular interactions (Figure 3) is one of many factors affecting molecular recognition processes. In the context of the HBeXB mentioned above, it is very interesting to see whether synergistic effects between halogen and hydrogen bonds (Figure 3, middle) can play a role in the case of the simple acyclic compounds.

Furthermore, the steric effects of the *ortho* substituent should also be mentioned, since these can have a significant impact on the extent to which the amide group participates in hydrogen bonding.

### 2.2. Synthesis of the Target Compounds and Crystal Structure of ***1***

**Scheme 1:**
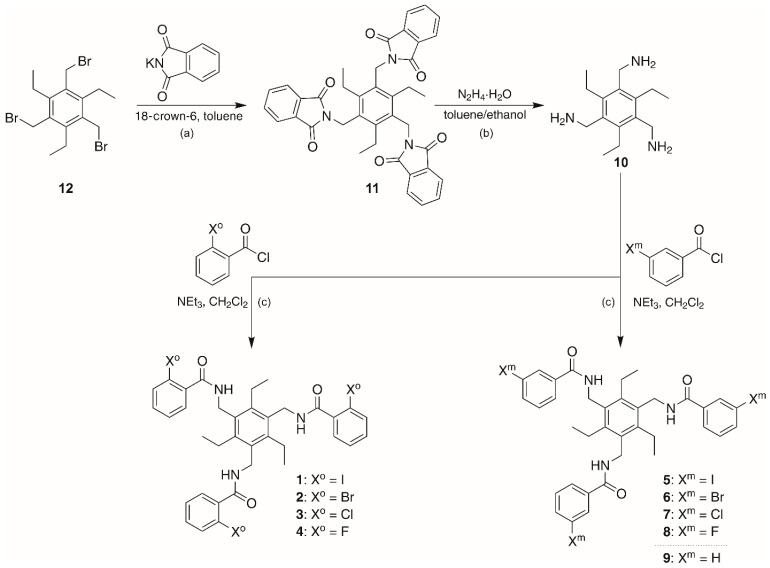
Synthesis of target compounds **1**–**9** starting from 1,3,5-tris(bromomethyl)-2,4,6-triethylbenzene (**12**). Reaction conditions: (a) potassium phthalimide, 18-crown-6, toluene, 100 °C; (b) N_2_H_4_·H_2_O, toluene/ethanol, 95 °C; (c) NEt_3_, CH_2_Cl_2_, rt (for details, see Section 4).

The target compounds **1**–**9** were synthesized from 1,3,5-tris(aminomethyl)-2,4,6-triethylbenzene (**10**), which was obtained from 1,3,5-tris(bromomethyl)-2,4,6-triethylbenzene (**12**) via Gabriel synthesis (Scheme 1). In a first step, the latter was reacted with potassium phthalimide and 18-crown-6 in toluene [37] to give the derivative **11** bearing phthalimidomethyl groups, which, in a second step, was converted to compound **10** by hydrazinolysis according to a procedure described in the literature [38], which was slightly modified. In this procedure, compound **11** was heated with five equivalents of hydrazine hydrate in a solvent mixture of toluene/ethanol 2:1 (*v*/*v*) at 95 °C for several hours (for details, see Section 4). Compound **10** was obtained in 99% yield and was reacted with differently substituted benzoic acid chlorides to give the desired products **1**–**9**. The reaction was carried out at room temperature in dichloromethane with triethylamine as base under inert gas and exclusion of light.

The reference compound **9** without halogen substituents, which has already been described in the literature [36], was synthesized for comparison purposes and examined with regard to its complexation properties.

Crystal structure of compound **1**

**Figure 4:**
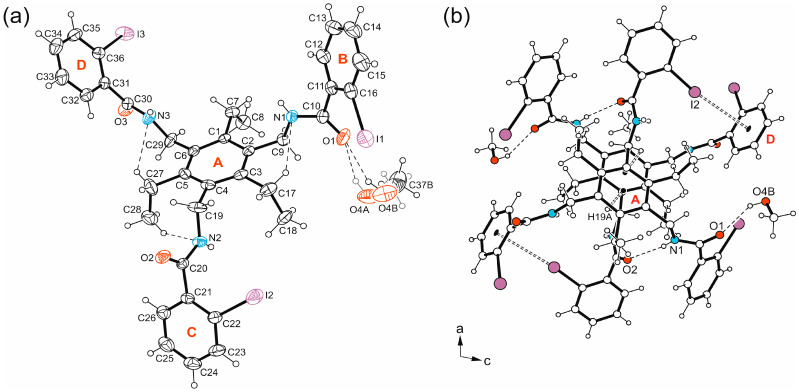
(**a**) Perspective view of the molecular structure of **1·**MeOH including the atom-numbering and ring specification. Displacement ellipsoids of the non-hydrogen atoms are drawn at the 50% probability level. (**b**) Structure of the dimer of the 1:1 host–guest unit. Hydrogen bonds are marked as dashed lines and arene-based interactions (C-I⋯π, C-H⋯π) as dashed double lines.

In the case of compound **1**, crystallization from methanol yields colourless blocks of the monoclinic space group *P*2_1_/*n*, which proved to be a methanol monosolvate of **1**. The asymmetric unit of the cell contains one molecule of **1** and one methanol molecule (see Figure 4a), the latter disordered over two positions (s.o.f. 0.68/0.32). The crystallographic data and geometric parameters for noncovalent interactions in the crystal structure of **1·**MeOH are summarized in Tables S1 and S2. To simplify the structural descriptions, the aromatic building blocks of the molecule have been labelled with capital letters in the figure showing the molecular structure.

In the crystal structure, the receptor molecule **1** exhibits a conformation in which the three functionalized side-arms adopt an *aab* arrangement with respect to the mean plane of the central arene ring. Taking the ethyl groups into account, the substituents attached to benzene ring follow an *ab’ab’bb’* arrangement (*a* = above, *b* = below, *b’* = ethyl below [39,40]). The aryl units (**B**–**D**) of the side-arms are inclined at angles of 62.7(2), 55.5(2) and 63.4(2)° to the plane of the benzene ring (**A**). These values together with the torsion angles of 140.8(3), −162.9(3) and 178.2(3)° formed by the atomic sequences C2-C9-N1-C10, C4-C19-N2-C20 and C6-C29-N3-C30 indicate the different degrees of twisting of the molecular arms. The conformation of the molecule appears to be stabilized by three intramolecular C-H⋯N bonds involving the H atoms of ethyl substituents [*d*(H⋯N) 2.55–2.61 Å].

As shown in Figure 4a, the methanol molecule is linked to the amide atom O1 of **1** by an O-H⋯O bond. An inversion-symmetric pair of these 1:1 host–guest units, held together via N-H⋯O=C bonds [*d*(H⋯O) 2.17(3) Å, ∢(N-H⋯O) 169(4)°] and weak C-I⋯π [*d*(I⋯*Cg*) 3.626(1) Å, ∢(C-I⋯*Cg*) 168.0(1)°] and C-H⋯π interactions [*d*(H⋯*Cg*) 2.80 Å, ∢(C-H⋯*Cg*) 121°] (see Figure 4b), represents the basic supramolecular unit of the present crystal structure.

**Figure 5:**
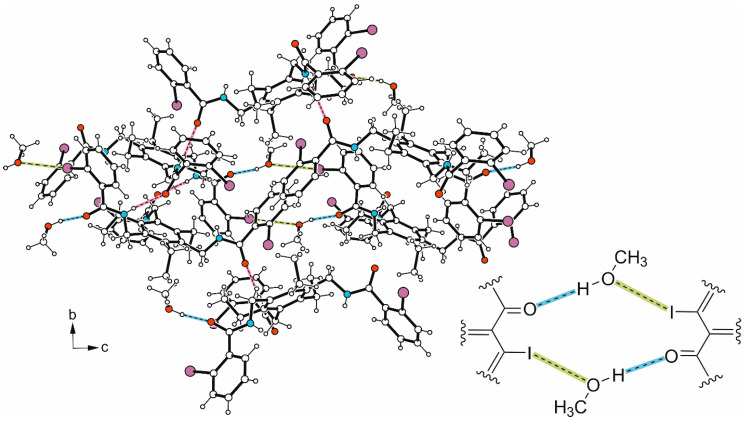
Excerpt of the crystal structure of **1·**MeOH showing the mode of noncovalent intermolecular bonding; O-H⋯O bonds are highlighted as light blue, N-H⋯O as pink and C-I⋯O bonds as yellow.

These dimeric units are in turn linked by further N-H⋯O bonds to form infinite strands that run in the *111* direction of the crystal structure. The remaining N-H hydrogen atom is excluded from molecular cross-linking. The linking of these one-dimensional molecular aggregates with each other occurs with the participation of the iodine atom I1 of the receptor and the O atom of the alcohol molecule via C-I⋯O bond formation [*d*(I⋯O) 3.531(18)Å, ∢(C-I⋯*Cg*) 154.4(3)°]. The excerpt of the crystal structure shown in Figure 5 and the packing diagram displayed in Figure S1 illustrate the mode of noncovalent bonding between the molecules.

### 2.3. Binding Studies

#### 2.3.1. Results of the ^1^H-NMR Spectroscopic Titrations

The complexation properties of compounds **1**–**9** were analyzed by ^1^H NMR titrations using octyl β-d-glucopyranoside (**βGlc**) as a binding partner, which is frequently used as a test substrate in studies on the molecular recognition of carbohydrates. The titrations were performed either with constant concentration of the corresponding triethylbenzene derivative and increasing concentration of **βGlc** or with constant sugar concentration and variable receptor concentration (inverse titration) in CDCl_3_ at 293 K. In addition, experiments were performed in water-containing CDCl_3_. The programmes WinEQNMR [41] and SupraFit [42] were used to analyze the ^1^H NMR titration data.

Furthermore, NMR-based studies on the influence of intramolecular interactions were also carried out, such as experiments using the method described by Abraham et al. [43,44,45,46,47] for the quantitative assessment of intramolecular hydrogen bonding. The experimental investigations were supported by molecular modelling studies and quantum chemical calculations.

The binding studies in CDCl_3_ showed some interesting relationships between structure and binding affinity for compounds **1**–**8**; however, all the tested compounds, as well as the reference compound **9**, are weak receptors for **βGlc** under the chosen titration conditions. It should also be noted that the differences in binding affinities are not very pronounced, but a clear trend is visible.

**Figure 6:**
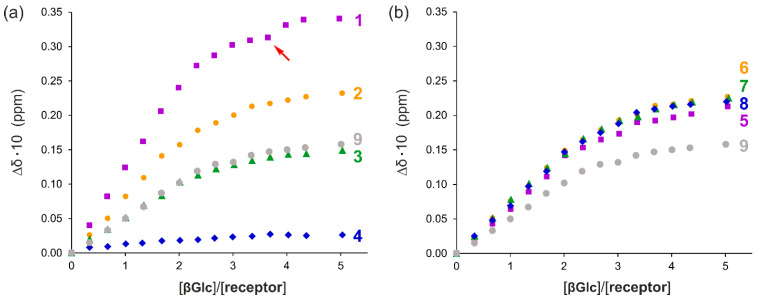
Changes in the chemical shifts in the NH signal after addition of 0.0 to 5.1 equivalents of β-d-glucopyranoside (**βGlc**) in the ^1^H NMR titration experiments (500 MHz, CDCl_3_, 293 K) for (**a**) compounds **1**–**4** with *ortho*-substituted benzoyl units and (**b**) the *meta*-substituted derivatives **5**–**8** (concentration of **1**–**9** was ~1 mmol). The results for compound **9** are shown as a reference in both diagrams. In the case of compound **1**, the change in chemical shifts observed after the addition of about 3.5 equivalents of **βGlc** (marked with a red arrow) was reproducible in repeated titrations and does not represent an artefact (this change is not as pronounced with the other derivatives).

Exemplary results of the titration experiments, in which the concentration of the triethylbenzene derivative was kept constant and that of the monosaccharide was varied, are presented in Figure 6. Shown are the complexation-induced changes in chemical shifts observed for the NH signal of **1**–**8**, which undergoes a downfield shift, indicating the involvement of NH in the formation of hydrogen bonds. The results obtained for **9** are shown for comparison purposes. In all cases, the complexation-induced shifts are small and indicate weak binding properties of the compounds with respect to the tested substrate.

It is easily recognizable that (as anticipated) the differences within the series of compounds **1**–**4** with the halogen substituent placed *ortho* to the CONH functionality (Figure 6a) are larger than those observed for the *meta*-substituted derivatives **5**–**8** (Figure 6b). Examples of plots of the experimental and calculated chemical shifts as a function of added octyl β-d-glucopyranoside (**βGlc**) are given in the Supporting Information.

For compounds **1**–**4** (Figure 6a), bearing the halogen substituent in the *ortho* position to the amide functionality, the binding affinity increases in the sequence **4** (*o*-F) < **3** (*o*-Cl) < **2** (*o*-Br) < **1** (*o*-I) (see Table 1).

The sequence mentioned above correlates with the strength of the halogen bond donor, but in principle, the formation of intramolecular NH⋯X hydrogen bonds is also conceivable, which would first have to be broken before complex formation with the substrate, and could also explain the trend. However, NMR-based investigations (see Section 2.3.3) indicate that these intramolecular hydrogen bonds are non-existent or very weak in the systems mentioned. Additional information was obtained by theoretical calculations, which are described later.

Another factor that should be pointed out is the effect of steric *ortho*-substitution of the aromatic ring in the case of **1**–**4**. The impact of this substitution on amide resonance and rotational barriers in common benzamides has been investigated [48]. Some studies support the use of *ortho*-substitution in benzamides to enhance amide resonance. Investigations on the influence of *ortho* substituents on the reactivity of benzamides, such as the rate of amide hydrolysis [49], showed that this is largely determined by the *ortho* steric effect.

In the case of the titrations of **βGlc** with the compounds **1** and **2** (inverse titrations), the upfield shifts in the sugar CHs indicate their involvement in CH···π interactions with the aromatic unit of the receptor (sugar–aromatic interactions; for examples of review articles on this topic, see refs. [50,51,52]; for examples of CH···π interactions in complexes of carbohydrates with artificial receptors, see refs. [53,54,55,56]). However, compared to the potent triethylbenzene-based receptors that we have previously studied (e.g., see refs. [57,58,59,60,61,62]), the complexation-induced chemical shifts are small, reflecting the weaker binding effectiveness of the receptors described here.

For compounds **5**–**8** with a halogen substituent in the *meta* position of the benzoylaminomethyl unit, the differences in the binding constants between the individual halogen derivatives are, as expected, minimal (see Table 1). Furthermore, the binding efficiencies of the iodo-, bromo- and chloro-substituted derivatives **5**–**7** are in accordance with expectations lower than those of the *ortho*-substituted analogues **1**–**3**.

**Figure 7:**
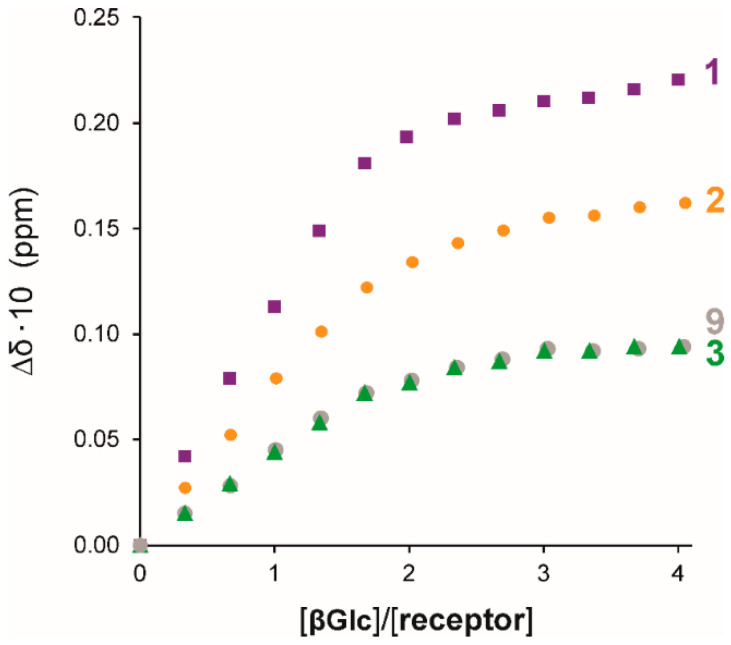
Changes in the chemical shifts in the NH signal after addition of 0.0 to 4.1 equivalents of β-d-glucopyranoside (**βGlc**) in the ^1^H NMR titration experiments [500 MHz, CDCl_3_/water (0.03 M), 293 K] for the iodo, bromo and chloro derivatives **1**–**3** with *ortho*-substituted benzoyl units. The results for compound **9** are shown as a reference.

The binding properties of the compounds containing *ortho*-substituted benzoylaminomethyl units, as well as the reference compound **9**, were also tested in the presence of small amounts of water (0.03 M) in CDCl_3_ by using ^1^H NMR titrations. The titration experiments, which were repeated three times for each compound, indicated an increase in binding strength in the presence of water (see Table 1). The increase observed for the iodo- and bromo-substituted derivatives **1** and **2** was larger (factors of 2.4 and 2.3) than that observed for the reference compound **9** (factor about 1.6). Both in experiments conducted in dry CDCl_3_ and in the presence of small amounts of water, the iodine- and bromine-substituted derivatives **1** and **2** proved to be more effective than the analogue **3** bearing the chlorine substituent (see Table 1 and Figure 7). Compared to **9**, the binding strength increased by a factor 2.6, 1.9 and 1.2 for the iodo, bromo and chloro derivative, respectively.

**Figure 8:**
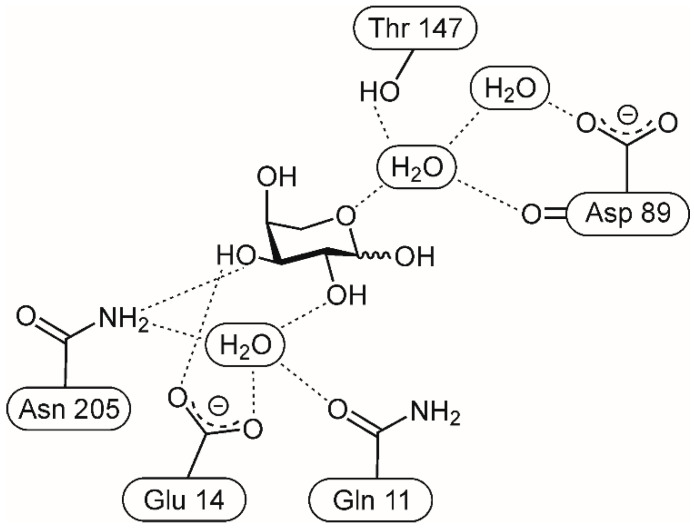
Examples of water-mediated hydrogen bonds in the complex of L-arabinose-binding protein (ABP) with α- or β-L-arabinose [4].

Positive effects of small amounts of water on binding strength have been reported for artificial carbohydrate receptors [53,63,64,65,66] and these effects have been attributed, among other things, to the possible formation of water-mediated hydrogen bonds, which are also frequently observed in the crystal structures of protein–carbohydrate complexes (for example, see Figure 8) [1,2,3,4,5,9].

The observed effect of the presence of water may also indicate the involvement of interactions with more pronounced hydrophobic character in the binding process. It should also be noted that, in addition to the water-mediated hydrogen bonds mentioned above, the importance of halogen–water–hydrogen bonds in molecular recognition processes has also been discussed in the literature (i.e., the participation of water molecules as bridges between halogen and hydrogen bonds) [67,68]. Analysis of the crystal structures of various biological systems revealed that water appears to play an important role in affecting the relationships between hydrogen and halogen bonds [26].

**Figure 9:**
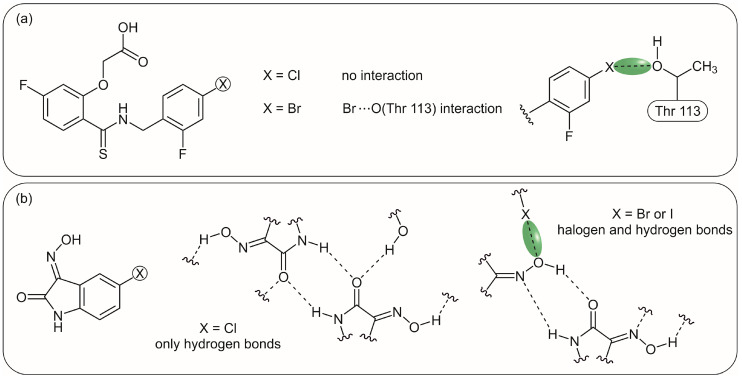
(**a**) Structure of an aldose reductase inhibitor (X = Br), whose activity is strongly affected by the formation of a C-Br⋯O interaction with Thr113 [69], (**b**) Structures of 5-halogeno-1*H*-isatin oximes and examples of hydrogen and halogen bonds in their crystal structures [70].

In the case of compounds tested here, the more positive effects of iodine and bromine (compounds **1** and **2**) compared to chlorine (compound **3**) are consistent with findings on halogen bond formation. Figure 9 shows two of many examples of other systems known from the literature that reflect this. Figure 9a illustrated the structure of an aldose reductase inhibitor (X = Br), the potency of which is affected by the formation of a C-Br⋯O interaction with Thr113 [69]. Such favourable interactions cannot be formed in the case of the chloro-substituted derivative. In the crystal structures of 5-halogeno-1*H*-isatin oximes (Figure 9b), C-X⋯O halogen bonds were only observed for the bromo- and iodo-substituted derivatives, but no halogen bonds were formed in the case of the chloro-substituted analogue. The chlorine was unable to compete with the hydrogen bond donors and to act as a halogen bond donor [70].

Despite the structural simplicity of the tested compounds, the relationships between the various interactions involved in the binding process are complex. However, the favourable effect expected from the *ortho*-substitution of the benzoylaminomethyl units can be observed, particularly with regard to the iodo and bromo substituents (compounds **1** and **2**).

#### 2.3.2. Statistical Analysis of the Titration Data

**Figure 10:**
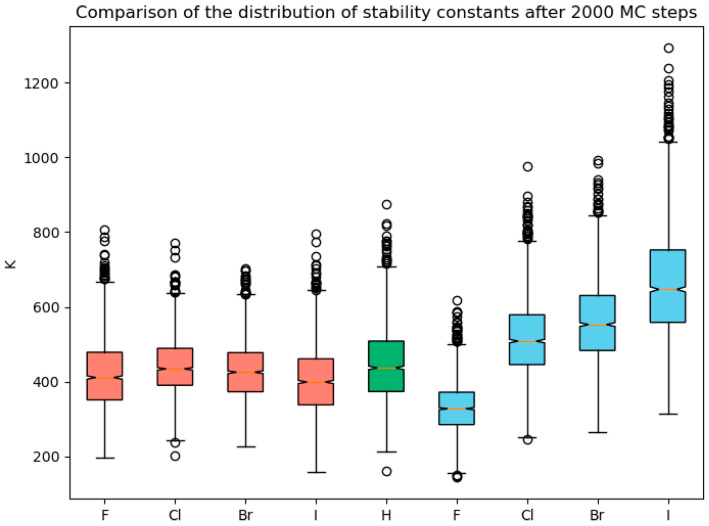
Boxplots of the stability constants obtained from Monte Carlo simulations for compounds **1**–**8** with the *meta-* (red, compounds **5**–**8**) and *ortho*-substituted (blue, **1**–**4**) benzoyl moieties (titrations in dry CDCl_3_). The boxplot for the reference compound **9** is shown in green.

In addition to the analysis of the ^1^H NMR titration data as described in Section 2.3.1, we performed a Monte Carlo simulation for compounds **1**–**9** using the best-fit parameters and the remaining SEy as the experimental error with 2000 steps, as implemented in SupraFit [41]. The resulting stability constants are plotted as boxplots (for titrations in dry CDCl_3_). Figure 10 shows the trend of increasing the binding constants for compounds with *ortho*-substituted benzoyl moieties **1**–**4** [**4** (*o*-F) < **9** (H) < **3** (*o*-Cl) < **2** (*o*-Br) < **1** (*o*-I)], shown as blue boxplots. The stability constants obtained for the compounds **5**–**8**, bearing a halogen substituent in the *meta* position (red boxplots), do not follow the same trend as the *ortho*-substituted analogues.

#### 2.3.3. Studies on the Formation of Intramolecular NH⋯X Hydrogen Bonds (Experimental Investigations and Quantum Chemical Calculations)

According to the Abraham method mentioned in Section 2.3.1, which involves the direct comparison of the chemical shifts in the NH signals in CDCl_3_ and DMSO [43,44,45,46], the difference in the chemical shift [∆δ = δ(DMSO)-δ(CDCl_3_)] can be converted into the acidity parameter A_NMR_ of the hydrogen bonds using the equation A_NMR_ = 0.0065 + 0.133·∆δ. The A_NMR_ can be used as a quantitative assessment of intramolecular hydrogen bonds in the corresponding compounds with NH functionality. If the NH group forms an intramolecular hydrogen bond, A_NMR_ is reduced because the tendency to form an intermolecular hydrogen bond decreases. Quantitatively, an A_NMR_ > 0.16 indicates that the NH group is not involved in an intramolecular hydrogen bond, whereas an A_NMR_ < 0.05 suggests that it is.

For compounds **1**–**4**, the following A_NMR_ values were obtained: 0.37 (**1**, *o*-I), 0.35 (**2**, *o*-Br), 0.33 (**3**, *o*-Cl) and 0.23 (**4**, *o*-F); for the reference compound **9**, an A_NMR_ value of 0.33 was determined. Table S3 in the Supporting Information shows the results obtained for compounds **1**–**4** and **9** as well as some examples of the application of the Abraham method using structures that are structurally related to the subunits of the target compounds investigated here.

The A_NMR_ values do not indicate the presence of intramolecular NH⋯X hydrogen bonds in the case of **1**–**4**. However, it should be noted that the A_NMR_ value for X = F is significantly lower than the values for the other derivatives, and considering the possible limitations of the Abraham method [47], this may be indicative of a very weak NH⋯F intramolecular hydrogen bond. The similarity of the A_NMR_ values for **1**–**3** to that of **9** speaks against the formation of intramolecular NH⋯X (X = Cl, Br, I) hydrogen bonds. Also, in the crystal structure of the iodine derivative **1** described above, the intramolecular NH⋯I hydrogen bond is not observed in any of the three (benzoylamino)methyl units, but this reflects the situation in the solid state, where packing effects play a role.

The (benzoylamino)methyl moiety represents a subunit of the compounds investigated here, and it should be noted that studies have been performed on the presence of an intramolecular NH⋯X hydrogen bond in benzamide derivatives bearing a halogen substituent in *ortho* position. In particular, some experimental and theoretical investigations on 2-fluorobenzamide derivatives (for example, *N*-phenyl derivative) have been reported, which provide indication for the presence of a weak intramolecular NH···F hydrogen bonding interaction [71,72,73,74]. It should be noted that the reported crystal structure of *N*-methyl-2-fluorobenzamide [75], with the fluorine atom disordered over two positions, indicates the formation of a weak intramolecular NH···F hydrogen bond in the solid state. Other interesting discussions on NH···F interactions in various molecules are described in references [73,74,76].

For comparison purposes with the literature data and to support the analysis of the binding mode of the compounds **1**–**4**, we also performed quantum chemical calculations with *N*-methyl- and *N*-benzyl-2-halogenobenzamides (for X = F, Cl, Br, I) as subunits of the compounds studied in this work. The calculations were carried out as described in the Section 4 and indicate that, only in the case of the fluorine derivatives, the formation of an intramolecular hydrogen bond is conceivable (see Supporting Information).

## 3. Conclusions

The 1,3,5-trisubstituted 2,4,6-triethylbenzene scaffold with subunits able to participate in the formation of hydrogen and halogen bonds was used as a model system to investigate the potential of halogenated building blocks for the construction of artificial carbohydrate receptors.

Initial binding studies using triethylbenzene derivatives with halogenated benzamidomethyl units and **βGlc** as a test substrate showed the expected influence of the type of halogen and its ring position on the binding properties of the investigated compounds. However, all tested compounds **1** to **8** as well as the reference compound **9** belong to weak receptors for **βGlc** under the chosen titration conditions.

As assumed, the differences in the binding constants between the individual halogen derivatives are more pronounced for compounds **1**–**4**, bearing a halogen substituent in the *ortho* position of the benzoylaminomethyl unit, than for the *meta*-substituted analogues **5**–**8**, for which the differences are almost negligible.

The binding efficiencies of the iodo-, bromo- and chloro-substituted derivatives **1**–**3** are in accordance with expectations higher than those of the *meta*-substituted analogues **5**–**7**. The binding affinity for the compounds first mentioned increases in the order **4** (*o*-F) < **3** (*o*-Cl) < **2** (*o*-Br) < **1** (*o*-I). In the presence of small amounts of water, an increase in binding affinity was observed, which was strongest for the iodo- and bromo-substituted derivatives **1** and **2** (factor 2.4 and 2.3, respectively, compared to measurements in dry CDCl_3_).

Although the binding affinity of **1**–**4** increases in an order, which correlates with the strength of the halogen bond donor, a clear statement about the involvement of halogen bonds in the binding process cannot be made on the basis of the present results. Despite the structural simplicity of the compounds, various factors influencing their binding properties must be considered (including the effect of steric *ortho*-substitution of the aromatic ring in the case of **1**–**4**, as mentioned in Section 2.3.1). The relationships between different interactions are complex, but a positive effect of the presence of iodine and bromine (compounds **1** and **2**) can be observed.

We are convinced that the incorporation of halogenated building blocks has great potential for the development of selective and efficient carbohydrate receptors that also function well in aqueous media. By optimizing the type of halogenated units and the receptor architecture, the construction of powerful carbohydrate receptors can be expected, which are also capable of engaging in halogen bond formation. The present studies are intended to provide impetus for the use of halogenated units in the development of new effective carbohydrate-binding agents.

## 4. Experimental Section

The benzoic acid chlorides used for the syntheses of the target compounds are commercially available (TCI) and were stored under argon. All solvents used for the syntheses were of minimum analytical reagent grade and dried. For the complexation studies, the tested compounds were previously dried under vacuum. Octyl β-d-glucopyranoside is commercially available (carbosynth, purity ≥ 99.5%).

**Synthesis of 1,3,5-Tris(phthalimidomethyl)-2,4,6-triethylbenzene (11)**

This reaction was carried out according to the procedure of Greenaway et al. [37].

**Synthesis of 1,3,5-Tris(aminomethyl)-2,4,6-triethylbenzene (10)**

A modification of the procedure described in the literature [38] was used for this reaction. 1,3,5-Tris(phthalimidomethyl)-2,4,6-triethylbenzene (**11**) (3.00 g, 4.70 mmol) was dissolved in 100 mL toluene/ethanol (2:1, *v*/*v*) and five equivalents of hydrazine hydrate were added. The reaction mixture was heated to 95 °C for 16 h and then the solvent was removed by distillation. The residue was suspended in 150 mL of toluene and 40% KOH solution (aq) was added with stirring until the solid was completely dissolved. The aqueous phase was separated and the organic phase was washed with a saturated aqueous solution of NaCl and dried over sodium sulphate. After removal of the solvent under vacuum, the product **10** was obtained as a white solid.

Yield 99% (1.17 g, 4.68 mmol); ^1^H-NMR (500 MHz, CDCl_3_): δ = 1.24 (t, 9H, *J* = 7.5 Hz), 2.83 (q, 6H, *J* = 7.5 Hz), 3.88 (s, 6H) ppm; ^13^C-NMR (125 MHz, CDCl_3_): δ = 16.9, 22.7, 39.8, 137.5, 140.5 ppm.

**General procedure for the synthesis of target compounds 1**–**9**

In a three-necked flask with reflux condenser and calcium chloride drying tube, 100 mg (0.40 mmol) of 1,3,5-tris(aminomethyl)-2,4,6-triethylbenzene (**10**) and triethylamine (3.5 equiv) were dissolved in dry dichloromethane (4–5 mL) under argon atmosphere. The corresponding benzoic acid chloride (3.5 equiv) was then slowly added in argon counterflow. In an exothermic reaction, a white solid precipitated, which dissolved after complete addition of the benzoyl chloride. The reaction mixture was stirred in the absence of light and under an argon atmosphere, and the progress of the reaction was monitored using TLC. After the reaction was completed, the mixture was separated by column chromato-graphy (SiO_2_, CH_2_Cl_2_/CH_3_CN, 8:1 *v*/*v*).

The reaction mixture was also processed in another way. First, about 5 mL of distilled water was added and the dichloromethane was removed under vacuum. The solid was then filtered off and washed with water and ice-cooled dichloromethane. After drying, the crude product was recrystallized from THF/hexanes (hexanes: mixture of isomers). The product was filtered off, washed with the cold solvent mixture and then dried under vacuum.

By using the chromatographic separation mentioned above, better yields were obtained than in the case of the alternative processing of the reaction mixture.

*1,3,5-Tris[(2-iodobenzoylamino)methyl]-2,4,6-triethylbenzene* (**1**): The reaction was carried out in 4 mL of dry dichloromethane; 2-iodobenzoyl chloride was dissolved in 2 mL of dichloromethane before being added to the reaction mixture. Yield 74% (279 mg, 0.30 mmol); m.p. 252 °C; ^1^H NMR (500 MHz, CDCl_3_): δ = 1.30 (t, 9H, *J* = 7.5 Hz), 2.89 (q, 6H, *J* = 7.5 Hz), 4.68 (d, 6H, *J* = 4.3 Hz), 5.53 (t, 3H, *J* = 4.3 Hz), 7.06–7.09 (m, 3H), 7.33–7.39 (m, 6H), 7.81–7.82 (m, 3H) ppm; ^13^C NMR (125 MHz, CDCl_3_): δ = 16.7, 23.5, 38.6, 92.4, 128.2, 128.3, 131.3, 131.7, 139.8, 141.9, 144.5, 169.0 ppm; HRMS (ESI): *m*/*z* calcd for C_36_H_36_I_3_N_3_O_3_ + Na^+^: 961.97829 [M + Na]^+^, found: 961.97867; elemental analysis calcd (%) for C_36_H_36_I_3_N_3_O_3_: C 46.03%, H 3.86%, N 4.47%; found: C 46.04%, H 3.86%, N 4.36%.

*1,3,5-Tris[(2-bromobenzoylamino)methyl]-2,4,6-triethylbenzene* (**2**): Yield 77% (246 mg, 0.30 mmol); m.p. 227–228 °C; ^1^H NMR (500 MHz, CDCl_3_): δ = 1.29 (t, 9H, *J* = 7.5 Hz), 2.85 (q, 6H, *J* = 7.5 Hz), 4.68 (d, 6H, *J* = 4.3 Hz), 5.76 (t, 3H, *J* = 4.1 Hz), 7.23–7.25 (m, 3H), 7.32–7.36 (m, 3H), 7.53–7.55 (m, 6H) ppm; ^13^C NMR (125 MHz, CDCl_3_): δ = 16.6, 23.2, 38.7, 119.1, 127.6, 129.6, 131.4, 131.7, 133.3, 137.4, 144.5, 167.2 ppm; HRMS (ESI): *m*/*z* calcd for C_36_H_36_Br_3_N_3_O_3_ + Na^+^: 822.01649 [M + Na]^+^, found: 822.01848; elemental analysis calcd (%) for C_36_H_36_Br_3_N_3_O_3_: C 54.16%, H 4.54%, N 5.26%; found: C 54.22%, H 4.57%, N 5.19%.

*1,3,5-Tris[(2-chlorobenzoylamino)methyl]-2,4,6-triethylbenzene* (**3**): Yield 67% (180 mg, 0.27 mmol); m.p. 229–230 °C; ^1^H NMR (500 MHz, CDCl_3_): δ = 1.28 (t, 9H, *J* = 7.5 Hz), 2.83 (q, 6H, *J* = 7.5 Hz), 4.69 (d, 6H, *J* = 4.3 Hz), 5.98 (t, 3H, *J* = 4.1 Hz), 7.30–7.37 (m, 9H), 7.66–7.68 (m, 3H) ppm; ^13^C NMR (125 MHz, CDCl_3_): δ = 16.5, 23.1, 38.8, 127.2, 130.2, 130.2, 130.5, 131.4, 131.7, 134.7, 144.5, 166.1 ppm; HRMS (ESI): *m*/*z* calcd for C_36_H_36_Cl_3_N_3_O_3_ + Na^+^: 688.16926 [M + Na]^+^, found: 688.16862; elemental analysis calcd (%) for C_36_H_36_Cl_3_N_3_O_3_: C 65.02%, H 5.46%, N 6.32%; found: C 64.94%, H 5.69%, N 6.17%.

*1,3,5-Tris[(2-fluorobenzoylamino)methyl]-2,4,6-triethylbenzene* (**4**): Yield 65% (160 mg, 0.26 mmol); m.p. 213–214 °C; ^1^H NMR (500 MHz, CDCl_3_): δ = 1.27 (t, 9H, *J* = 7.5 Hz), 2.82 (q, 6H, *J* = 7.5 Hz), 4.73 (d, 6H, *J* = 3.7 Hz), 6.57–6.60 (m; 3H), 7.05–7.09 (m, 3H), 7.23–7.29 (m, 3H), 7.44–7.48 (m, 3H), 8.11–8.15 (m, 3H) ppm; ^13^C NMR (125 MHz, CDCl_3_): δ = 16.4, 23.1, 38.7, 115.9, 120.8, 124.9, 132.0, 133.3, 144.6, 159.6, 161.5, 163.0 ppm; HRMS (ESI): *m*/*z* calcd for C_36_H_36_F_3_N_3_O_3_ + Na^+^: 638.26010 [M + Na]^+^, found: 638.25848; elemental analysis calcd (%) for C_36_H_36_F_3_N_3_O_3_: C 70.23%, H 5.89%, N 6.82%; found: C 70.14%, H 5.93%, N 6.74%.

*1,3,5-Tris[(3-iodobenzoylamino)methyl]-2,4,6-triethylbenzene* (**5**): Yield 53% (200 mg, 0.22 mmol); m.p. 241–242 °C; ^1^H NMR (500 MHz, CDCl_3_): δ = 1.28 (t, 9H, *J* = 7.3 Hz), 2.81 (q, 6H, *J* = 7.5 Hz), 4.70 (d, 6H, *J* = 4.3 Hz), 5.90 (t, 3H, *J* = 4.3 Hz), 7.14–7.17 (m, 3H), 7.70–7.72 (m, 3H), 7.81–7.83 (m, 3H), 8.04–8.05 (m, 3H) ppm; ^13^C NMR (125 MHz, CDCl_3_): δ = 16.6, 23.3, 38.7, 94.3, 126.2, 130.4, 132.1, 135.8, 136.0, 140.6, 144.8, 165.6 ppm; HRMS (ESI): *m*/*z* calcd for C_36_H_36_I_3_N_3_O_3_ + Na^+^: 961.97829 [M + Na]^+^, found: 961.97612; elemental analysis calcd (%) for C_36_H_36_I_3_N_3_O_3_: C 46.03%, H 3.86%, N 4.47%; found: C 46.13%, H 3.84%, N 4.52%.

*1,3,5-Tris[(3-bromobenzoylamino)methyl]-2,4,6-triethylbenzene* (**6**): Yield 60% (192 mg, 0.24 mmol); m.p. 236 °C; ^1^H NMR (500 MHz, CDCl_3_): δ = 1.28 (t, 9H, *J* = 7.5 Hz), 2.81 (q, 6H, *J* = 7.5 Hz), 4.70 (d, 6H, *J* = 3.9 Hz), 5.90 (t, 3H, *J* = 3.9 Hz), 7.28–7.31 (m, 3H), 7.61–7.63 (m, 3H), 7.67–7.68 (m, 3H), 7,83–7.86 (m, 3H) ppm; ^13^C NMR (125 MHz, CDCl_3_): δ = 16.6, 23.3, 38.8, 122.8, 125.6, 130.0, 130.3, 132.1, 134.7, 135.9, 144.8, 165.7 ppm; HRMS (ESI): *m*/*z* calcd for C_36_H_36_Br_3_N_3_O_3_ + Na^+^: 822.01649 [M + Na]^+^, found: 822.01415; elemental analysis calcd (%) for C_36_H_36_Br_3_N_3_O_3_: C 54.16%, H 4.54%, N 5.26%; found: C 54.25%, H 4.51%, N 5.27%.

*1,3,5-Tris[(3-chlorobenzoylamino)methyl]-2,4,6-triethylbenzene* (**7**): Yield 70% (185 mg, 0.28 mmol); m.p. 223 °C; ^1^H NMR (500 MHz, CDCl_3_): δ = 1.28 (t, 9H, *J* = 7.5 Hz), 2.82 (q, 6H, *J* = 7.5 Hz), 4.70 (d, 6H, *J* = 4.4 Hz), 5.92 (t, 3H, *J* = 4.4 Hz), 7.34–7.37 (m, 3H), 7.45–7.48 (m, 3H), 7.61–7.63 (m, 3H), 7.69–7.70 (m, 3H) ppm; ^13^C NMR (125 MHz, CDCl_3_): δ = 16.6, 23.3, 38.7, 125.1, 127.1, 130.0, 131.8, 132.1, 134.8, 135.7, 144.7, 165.8 ppm; HRMS (ESI): *m*/*z* calcd for C_36_H_36_Cl_3_N_3_O_3_ + Na^+^: 688.16926 [M + Na]^+^, found: 688.16810; elemental analysis calcd (%) for C_36_H_36_Cl_3_N_3_O_3_: C 65.02%, H 5.46%, N 6.32%; found: C 64.78%, H 5.51%, N 6.24%.

*1,3,5-Tris[(3-fluorobenzoylamino)methyl]-2,4,6-triethylbenzene* (**8**): Yield 62% (154 mg, 0.25 mmol); m.p. 254 °C; ^1^H NMR (500 MHz, CDCl_3_): δ = 1.27 (t, 9H, *J* = 7.5 Hz), 2.82 (q, 6H, *J* = 7.5 Hz), 4.70 (d, 6H, *J* = 4.4 Hz), 5.94 (t, 3H, *J* = 4.3 Hz), 7.17–7.21 (m, 3H), 7.36–7.40 (m, 3H), 7.43–7.46 (m, 3H), 7.47–7.49 (m, 3H) ppm; ^13^C NMR (125 MHz, CDCl_3_): δ = 16.6, 23.2, 38.7, 114.2, 118.7, 122.3, 130.3, 132.1, 136.2, 144.7, 161.7, 163.7, 165.8 ppm; HRMS (ESI): *m*/*z* calcd for C_36_H_36_F_3_N_3_O_3_ + Na^+^: 638.26010 [M + Na]^+^, found: 638.25789; elemental analysis calcd (%) for C_36_H_36_F_3_N_3_O_3_: C 70.23%, H 5.89%, N 6.82%; found: C 70.06%, H 5.92%, N 6.86%.

*1,3,5-Tris[(benzoylamino)methyl]-2,4,6-triethylbenzene* (**9**): The reaction was carried out in 7 mL dry dichloromethane. Yield 58% (130 mg, 0.23 mmol); m.p. 263–264 °C; ^1^H NMR (500 MHz, CDCl_3_): δ = 1.28 (t, 9H, *J* = 7.4 Hz), 2.83 (q, 6H, *J* = 7.5 Hz), 4.71 (d, 6H, *J* = 4.3 Hz), 5.95 (t, 3H, *J* = 4.3 Hz), 7.39–7.42 (m, 6H), 7.48–7.51 (m, 3H), 7.73–7.75 (m, 6H) ppm; ^13^C NMR (125 MHz, CDCl_3_): δ = 16.6, 23.2, 38.7, 126.9, 128.6, 131.7, 132.3, 134.0, 144.6, 167.2 ppm; HRMS (ESI): *m*/*z* calcd for C_36_H_39_N_3_O_3_ + Na^+^: 584.28836 [M + Na]^+^, found: 584.28908.

**Quantum chemical calculations.** The calculated structures (*N*-methyl- and *N*-benzyl-2-halogenobenzamides) were drawn with avogadro [77] version 1.99 and pre-optimized using the GFN2-xTB method [78]. A subsequent conformational search was performed with crest 2.0 [79] and the GFN2-xTB method. Using ORCA 6 [80], the resulting conformations were further optimized using the r^2^SCAN-3c composite method [81,82,83,84] and in the geometry of the most stable conformation of each structure at r^2^SCAN-3c level, the NH···X distance and the dihedral angle characterizing the C_Ar_(X)C_Ar_C(O)NH plane were measured. To compensate for the varying covalent and van der Waals radii of each element, the individual covalent [85] and van der Waals [86] values of X (H, F, Cl, Br and I) were subtracted from the measured distances.

**Crystal structure analysis.** Crystals suitable for X-ray diffraction were obtained by slow evaporation of a methanol solution of **1**. These crystals were identified under a microscope, mounted on a glass fibre and analyzed in φ-scans with STOE equipment (image plate system IPDS-2) at 173 K, employing Mo-K_α_ radiation (λ = 0.71073 Å) monochromatized by graphite. Following data collection, indexing and integration with the program X-AREA [87], data reduction including scaling and absorption correction was carried out via the X-RED32 and LANA program suite [88,89]. Preliminary structure solutions were obtained by direct methods (SHELXT2014/5 [90]) employing the program XStep 32 [91] and then refined by full-matrix least-squares calculations based on F^2^ for all reflections using the program SHELXL-2019/2 [92]. Aprotic hydrogen atoms were refined using a riding model, while protic hydrogen atoms were identified from the electron density map and restrained to lie within common distances from their covalently bound O-/N-atoms (0.84/0.89 Å, respectively). All safe riding hydrogen atoms were refined anisotropically. The graphical representation of the molecular structures was performed using the programs ORTEP-III [93] and XP in SHELXTL [94]. Crystallographic data for the structure in this paper have been deposited with the Cambridge Crystallographic Data Centre as supplementary publication number CCDC 2479922.

## Figures and Tables

**Table 1:** Association constants for the complexation of **βGlc** with compounds **1**–**9** ^a–c^.

Compound	*o*-X	*K*_a_ [M^−1^] ^a–d^		Compound	*m*-X	*K*_a_ [M^−1^] ^a–c^ CDCl_3_
		CDCl_3_	CDCl_3_/Water (0.03 M)			
**1**	*o*-I	760	1820	**5**	*m*-I	397
**2**	*o*-Br	600	1380	**6**	*m*-Br	425
**3**	*o*-Cl	505	890	**7**	*m*-Cl	437
**4** ^d^	*o*-F	320	--	**8**	*m*-F	410
-----------	---------	---------	--------			
**9** ^e^	H	435	700			

^a^ Determined by ^1^H NMR spectroscopic titrations (500 MHz, 293 K) in dry CDCl_3_ and in the presence of water (0.03 M in CDCl_3_). ^b^ Very good reproducibility of the *K*_a_ values in triplicate titrations; for examples of fitting curves, see Supporting Information. ^c^ The standard deviation was ≤2%. ^d^ The *K*_a_ value for **4** was determined only on the basis of the complexation-induced chemical shifts in the CH signals of **4**. ^e^ The *K*_a_ value previously reported in the literature for compound **9** was determined under different experimental conditions [*K*_a_ = 710 M^−1^, 300 K, CDCl_3_ (dryness of the solvent unknown)] [36].

## Data Availability

The original contributions presented in this study are included in the article/Supplementary Material. Further inquiries can be d.irected to the corresponding author.

## Supplementary Materials

The following supporting information can be downloaded at: https://www.mdpi.com/article/10.3390/molecules31081237/s1, Crystallographic data for the methanol solvate of **1** (**1·**MeOH) (Tables S1 and S2, Figure S1). Abraham et al. method [43,44,45,46] for the quantitative assessment of intramolecular hydrogen bonding (Table S3). Quantum chemical calculations (Figures S2 and S3, Table S4). Description of the binding studies (Table S5). Examples of fitting curves for ^1^H NMR titrations (Figures S4–S7). ^1^H and ^13^C NMR spectra of compounds **1**–**9** (Figures S8–S25).
