# Supplementary material for: Use of Halogenated Units for the Construction of Artificial Carbohydrate Receptors
Source: Molecules. 2026 Apr 9;31(8):1237. doi: 10.3390/molecules31081237 (PMC13118432; doi:10.3390/molecules31081237)
Supplement: Supplementary file 1 [file molecules-31-01237-s001.zip › molecules-4171443-supplementary.pdf]

Supporting Information

Use of Halogenated Units for the Construction of Artificial Carbohydrate Receptors

Betty Fuhrmann, Conrad Hübler and Monika Mazik*

Institut für Organische Chemie, Technische Universität Bergakademie Freiberg, Leipziger Strasse 29, 09599 Freiberg, Germany

*Correspondence: monika.mazik@chemie.tu-freiberg.de; Tel.: +49-3731392389; Fax: +49-3731393170

1. Crystallographic data for the methanol solvate of **1** (**1**·MeOH) (Tables S1 and S2, Figure S1)
2. Abraham *et al.* method for the quantitative assessment of intramolecular hydrogen bonding (Table S3)
3. Quantum chemical calculations (Figures S2 and S3, Table S4)
4. Description of the binding studies (Table S5)
5. Examples of fitting curves for ^{1}H NMR titrations (Figures S4-S7)
6. ^{1}H and ^{13}C NMR spectra of compounds**1**–**9** (Figures S8-S25).

1. Crystal structure of the methanol solvate of 1 (1·MeOH).

Table S1. Crystallographic data for **1**·MeOH.

	**1·CH_3OH**
Empirical formula	$C_{37}H_{40}I_3N_3O_4$
Moiety formula	$C_{36}H_{36}I_3N_3O_3$, CH_4O
Formula weight	971.42
Crystal system	monoclinic
Space group	$P2_1/n$
a [Å]	13.6140(3)
b [Å]	17.2543(4)
c [Å]	16.1140(4)
α [°]	90
β [°]	106.229(2)
γ [°]	90
V [Å^3]	3634.35(15)
Z	4
F(000)	1896
D_c[Mg m^{-3}]	1.775
μ [mm^{-1}]	2.623
Temperature [K]	173(2)
No. of collected reflections	51203
within the *Θ*-limit [°]	1.32–28.40
Index ranges ±*h*, ±*k*, ±*l*	-17/16, -22/22, -20/20
No. of unique reflections	8353
R_{int}	0.054
Weighting expression *w*[a]	$[\sigma^2(F_o^2)+(0.0262P)^2+7.2310P]^{-1}$
No. of refined parameters	464
No. of restraints	14
No. of F values used ($I>2\sigma(I)$)	6986
R $(=\Sigma\|\Delta F\|/\Sigma\|F_o\|)$	0.034
wR on F^2	0.074
S (=Goodness of fit on F^2)	1.086
Final $\Delta\rho_{max}/\Delta\rho_{min}$ [e Å^{-3}]	0.70, -0.72

[a] $P=(F_o^2+2F_c^2)/3$

Table S2. Geometric parameters for noncovalent interactions in the crystal structure of **1**·MeOH.

Atoms		Distance (Å)		Angle (°)
D-H···A		D···A	H···A	D-H···A
C-I···O		C···O	I···O	C-I···O
C-I···π		C···I	C···*Cg*	C-I···*Cg*
C-H···π		C···*Cg*	H···*Cg*	D-H···*Cg*
N(1)-H(1)···O(2)	1-*x*, 1-*y*, 1-*z*	2.899(4)	2.05(3)	169(4)
N(2)-H(2)···O(3)	-0.5+*x*, 1.5-*y*, -0.5+z	3.009(4)	2.17(3)	162(4)
O(4B)-H(1B)···O(1)	*x*, *y*, *z*	2.877(18)	2.13(9)	149(10)
C(9)-H(9A)···O(1)	*x*, *y*, *z*	2.773(4)	2.39	102
C(17)-H(17B)···N(1)	*x*, *y*, *z*	3.316(5)	2.59	130
C(27)-H(27A)···N(3)	*x*, *y*, *z*	3.334(5)	2.61	130
C(28)-H(28A)···N(2)	*x*, *y*, *z*	3.370(5)	2.55	142
C(16)-I(1)···O(4B)[a]	1-*x*, 1-*y*, *z*	4.932(16)	3.531(18)	154.4(3)
C(12)-H(12)···*Cg*(C)[a]	1+*x*, *y*, *z*	3.392(4)	2.87	115
C(19)-H(19A)···*Cg*(A)[a]	1-*x*, 1-*y*, 1-z	3.417(4)	2.80	121
C(36)-I(3)···*Cg*(A)[a]	0.5+*x*, 1.5-*y*, 0.5+z	5.237(3)	3.504(1)	137.3(1)
C(22)-I(2)···*Cg*(D)[a]	1-*x*, 1-*y*, 1-z	5.698(3)	3.626(1)	168.0(1)
C(12)-H(12)···*Cg*(C)[a]	1+*x*, *y*, *z*	3.392(4)	2.87	115
C(19)-H(19A)···*Cg*(A)[a]	1-*x*, 1-*y*, 1-z	3.417(4)	2.80	121

[a]*Cg* means the centroid (centre of gravity) of the aromatic ring.
Ring A: C(1)…C(6); ring B: C(21)…C(26); ring A: C(31)…C(36).

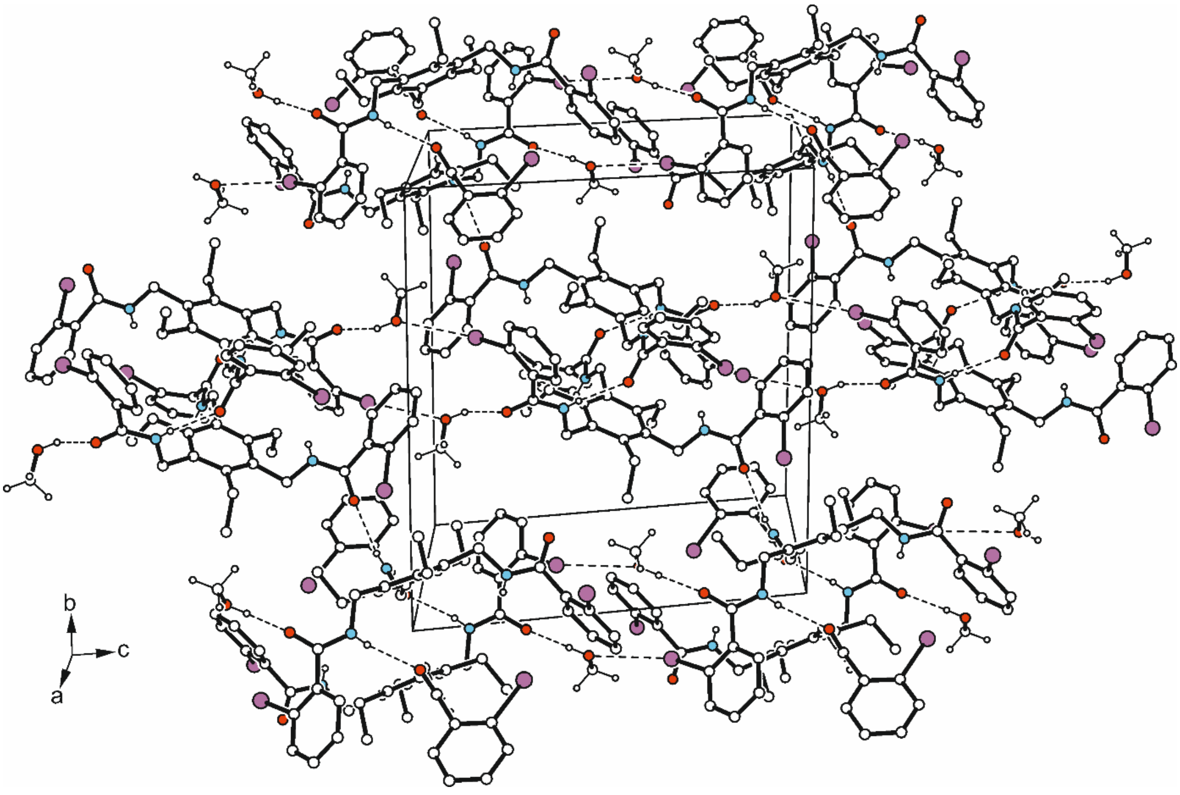

Figure S1. Packing structure of **1**·MeOH; intermolecular interactions are marked as dashed lines.

2. Abraham *et al.* method for the quantitative assessment of intramolecular hydrogen bonding.

Table S3. A_{NMR} values determined on the basis of the method according to Abraham *et al.* for some compounds known from the literature (top) and compounds analyzed in this work (bottom).

**Compound**	**A_{NMR}**	**ref.**
benzamide (H_b)	0.25	[A, B]
benzamide (H_a)	0.17	[C]
2-fluorobenzamide (H_b)	0.21	[C]
2-fluorobenzamide (H_a)	**0.13**	[C]
2-chlorobenzamide (H_b)	0.20	[D]
2-chlorobenzamide (H_a)	**0.16**	[D]
**1**	0.37	
**2**	0.35	
**3**	0.33	
**4**	0.23	
**9**	0.33	

In the case of the benzamides (refs. A-D), the determined A_{NMR} values indicate the presence of a weak intramolecular NH···X hydrogen bonding in only two cases.

The references A-D are cited in the manuscript as references [43-46].

[A] R. J. Abraham, L. Griffiths, M. Perez, *Magn. Reson. Chem.* **2013**, *51*, 143.

[B] R. J. Abraham, L. Griffiths, M. Perez, *Magn. Reson. Chem.* **2014**, *52*, 395.

[C] R. J. Abraham, J. J. Byrne, L. Griffiths, M. Perez, *Magn. Reson. Chem.* **2006**, *44*, 491.

[D] M. H. Abraham, R. J. Abraham, W. E. Acree, A. E. Aliev, A. J. Leo, W. L. Whaley, *J. Org. Chem.* **2014**, *79*, 11075.

3. Quantum chemical calculations

a) *N*-Methyl-2-halogenobenzamides

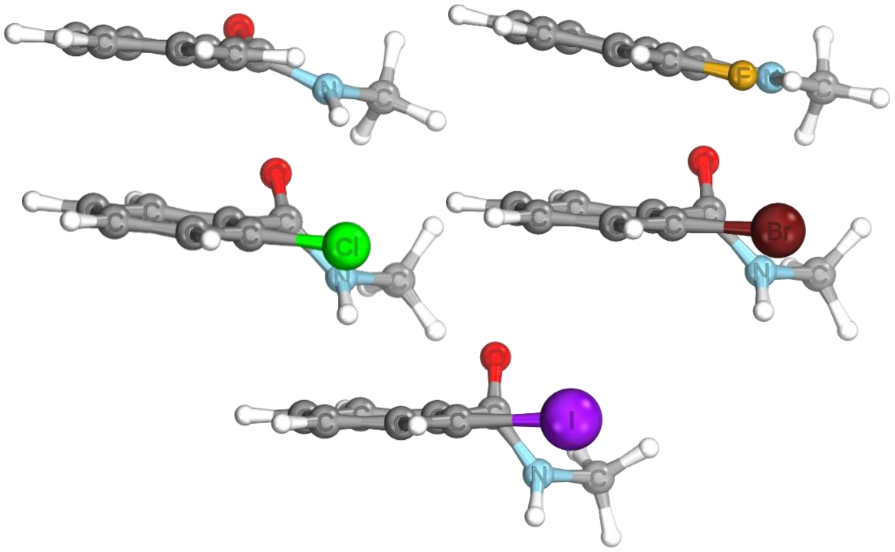

b) *N*-Benzyl-2-halogenobenzamides

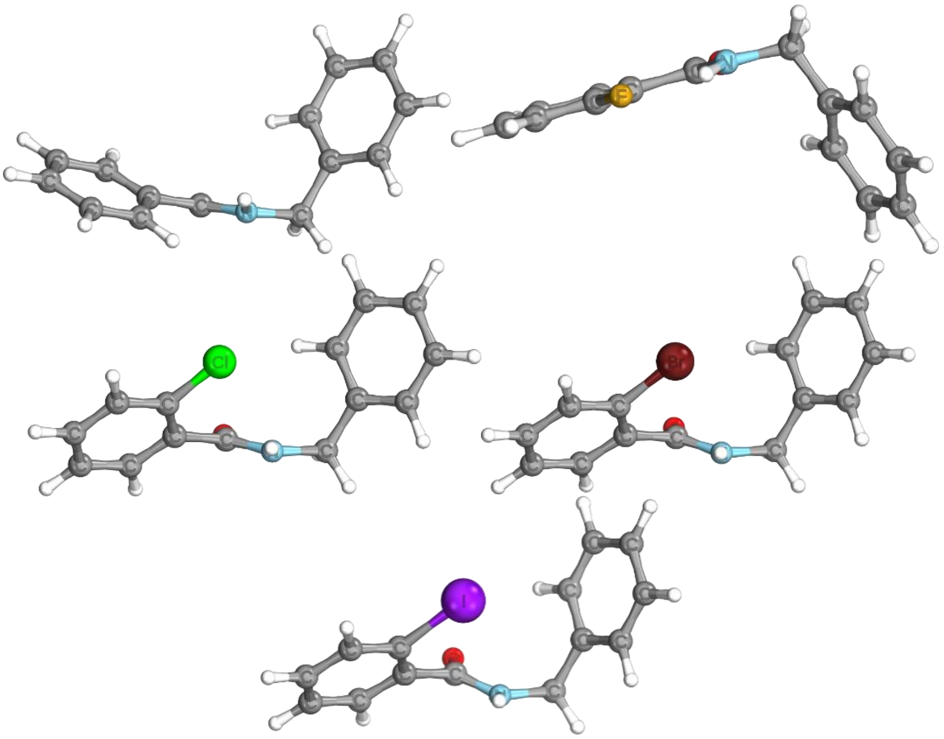

Figure S2. *N*-Methyl- and *N*-benzyl-2-halogenobenzamide structures (X = F, Cl, Br and I), optimized at TPSSH/def2-QZVPP level [*N*-methyl- and *N*-benzylbenzamides (X = H) are also considered]. A planar geometry for the $C_{Ar}(X)C_{Ar}C(O)NH$ unit which is favourable for the formation of the intramolecular NH···X hydrogen bond can only be observed for the fluorine derivatives.

Table S4: Dihedral angle and NH···X distances obtained for the most stable geometries of the *N*-methyl- and *N*-benzyl-2-halogenobenzamides in $CHCl_3$. Only for the fluorine derivates, a planar geometry alongside with the shortest X-H contact was observed.

	N-methyl-2-*halogen*obenzamides				*N*-benzyl-2-*halogen*obenzamides			
X	Dihedral angle [°]	r [Å]	r -r_{cov}^{X}[Å]	r -r_{vdw}^{X}[Å]	Dihedral angle [°]	[A]	r -r_{cov}^{X}[Å]	r -r_{vdw}^{X}[Å]
H	33.6	2.19	1.88	1.09	34.6	2.19	1.88	1.09
F	−2.6	1.96	1.39	0.49	−2.0	1.97	1.40	0.50
Cl	41.1	2.56	1.54	0.81	47.2	2.63	1.61	0.88
Br	45.8	2.69	1.49	0.86	50.2	2.75	1.55	0.92
I	55.6	2.99	1.60	1.01	54.4	2.95	1.56	0.97

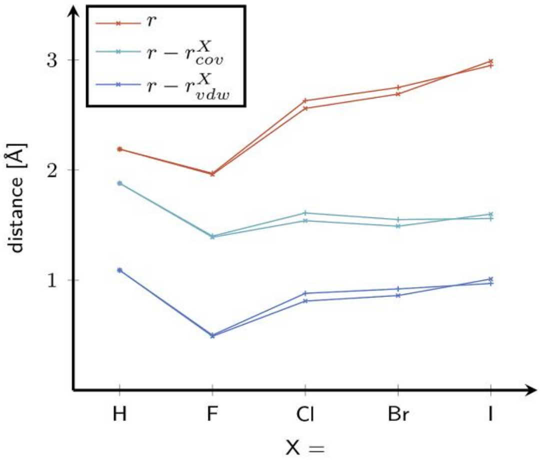

Figure S3. Evaluated NH···X distances for *N*-methyl and *N*-benzyl-2-halogenobenzamides optimised using TPSSH/def2-QZVPP in $CHCl_3$. The methyl and benzyl groups do not substantially affect the distance; the closest interaction is noted for X = F, whilst the distances are greater for other substituents, regardless of the element's inherent size.

4. Description of the binding studies

The ^{1}H NMR titrations were carried out in $CDCl_3$ or $CDCl_3$ containing defined amounts of water at 20 °C ($CDCl_3$ was deacidified over basic aluminium oxide [Brockmann I] and stored over molecular sieves). The $CDCl_3$/water mixtures were previously homogenized and degassed in an ultrasonic bath. Stock solutions in the respective solvent were prepared and homogenized for the corresponding receptor and sugar. These solutions and the solvent were added together in a manner that the concentration of the receptor was kept constant and that of the sugar was varied (titrations in which the concentration of the sugar was kept constant and that of the receptor was varied were also performed). The receptor concentration was adjusted to about 1mM to avoid self-aggregation. For each titration, 15-20 samples were prepared (for an example, see Table S5), thoroughly homogenized and the ^{1}H NMR spectra were recorded.

Table S5. ^{1}H NMR titration of compound **3** with octyl-β-D-glucopyranoside in $CDCl_3$.

	V_{rec}[mL]	c_{rec} [mM]	V_{sug}[mL]	c_{sug} [mM]	V_{solv}[mL]	ratio rec:sug
1	0.4	1.00	0.00	0.00	0.30	1:0.00
2	0.4	1.00	0.02	0.33	0.28	1:0.33
3	0.4	1.00	0.04	0.66	0.26	1:0.66
4	0.4	1.00	0.06	1.00	0.24	1:0.99
5	0.4	1.00	0.08	1.33	0.22	1:1.33
6	0.4	1.00	0.10	1.66	0.20	1:1.66
7	0.4	1.00	0.12	1.99	0.18	1:1.99
8	0.4	1.00	0.14	2.33	0.16	1:2.32
9	0.4	1.00	0.16	2.66	0.14	1:2.65
10	0.4	1.00	0.18	2.99	0.12	1:2.98
11	0.4	1.00	0.20	3.32	0.10	1:3.32
12	0.4	1.00	0.22	3.65	0.08	1:3.65
13	0.4	1.00	0.24	3.99	0.06	1:3.98
14	0.4	1.00	0.26	4.32	0.04	1:4.32
15	0.4	1.00	0.30	4.98	0.00	1:4.97

rec =receptor, sug = sugar, solv = solvent.

5. Examples of fitting curves for ^{1}H NMR titrations

5.1. Examples of fitting curves for ^{1}H NMR titrations (*ortho*-substituted derivatives)

a) b)

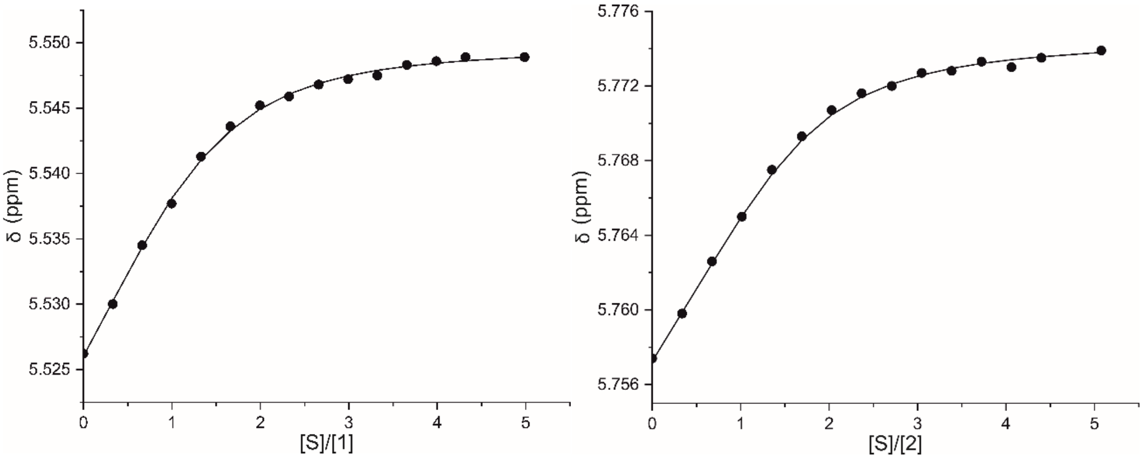

Figure S4. Plots of the experimental (points) and calculated (line) chemical shifts of **1** (a) and **2** (b) as a function of added octyl-β-D-glucopyranoside (βGlc) in $CDCl_3$/water (0.03 M).

a) b)

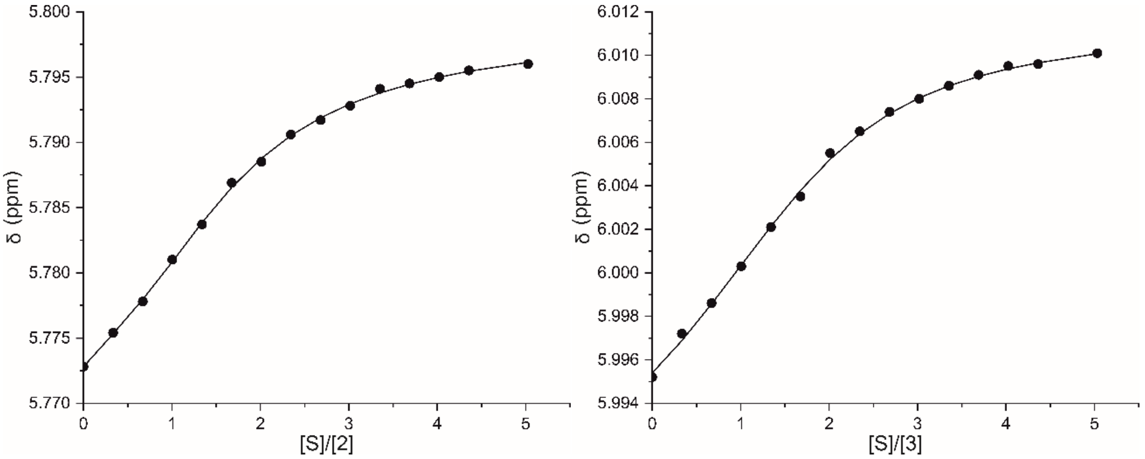

Figure S5. Plots of the experimental (points) and calculated (line) chemical shifts of **2** (a) and **3** (b) as a function of added octyl-β-D-glucopyranoside (βGlc) in $CDCl_3$ (1:1/1:2 receptor-sugar binding model; the K_{11} values are given in Table 1 in the manuscript, the K_{12} values are only in the range of 10^1).

5.2. Examples of fitting curves for ^{1}H NMR titrations (*meta*-substituted derivatives)

a) b)

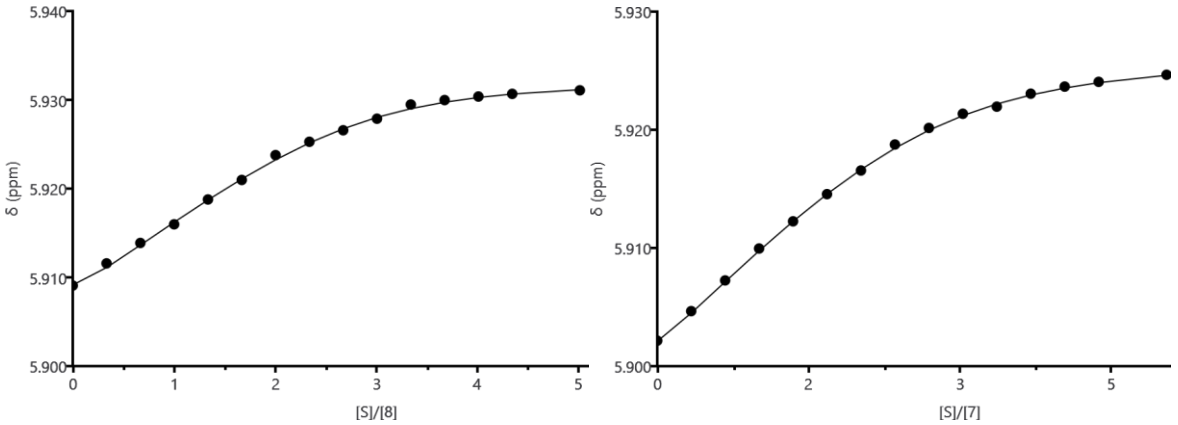

Figure S6. Plots of the experimental (points) and calculated (line) chemical shifts of **8** (a) and **7** (b) as a function of added octyl-β-D-glucopyranoside (βGlc) in $CDCl_3$.

a) b)

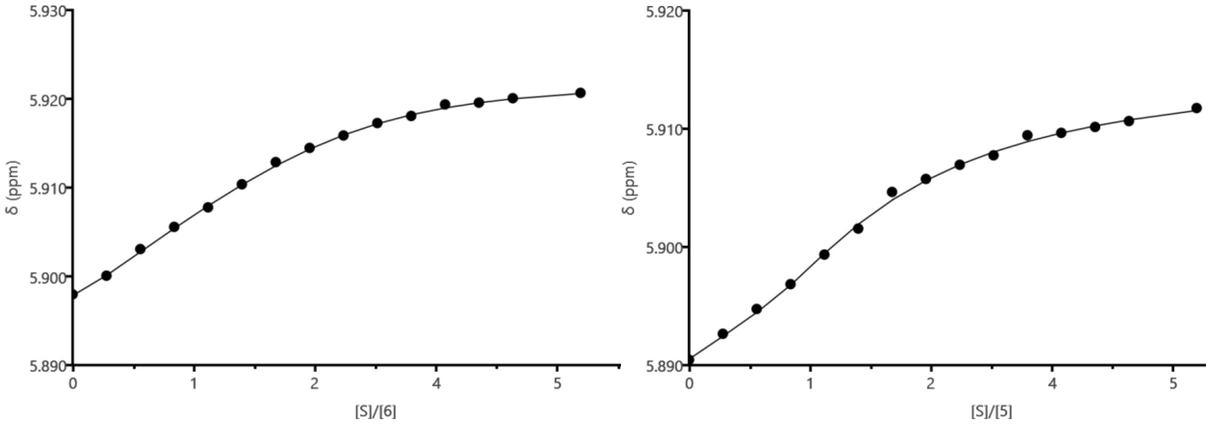

Figure S7. Plots of the experimental (points) and calculated (line) chemical shifts of **6** (a) and **5** (b) as a function of added octyl-β-D-glucopyranoside (βGlc) in $CDCl_3$ (1:1/1:2 receptor-sugar binding model; the K_{11} values are given in Table 1 in the manuscript, the K_{12} values are only in the range of 10^1).

6. ^{1}H und ^{13}C NMR spectra of compounds 1–9

6.1 ^{1}H and ^{13}C NMR spectra of compound 1

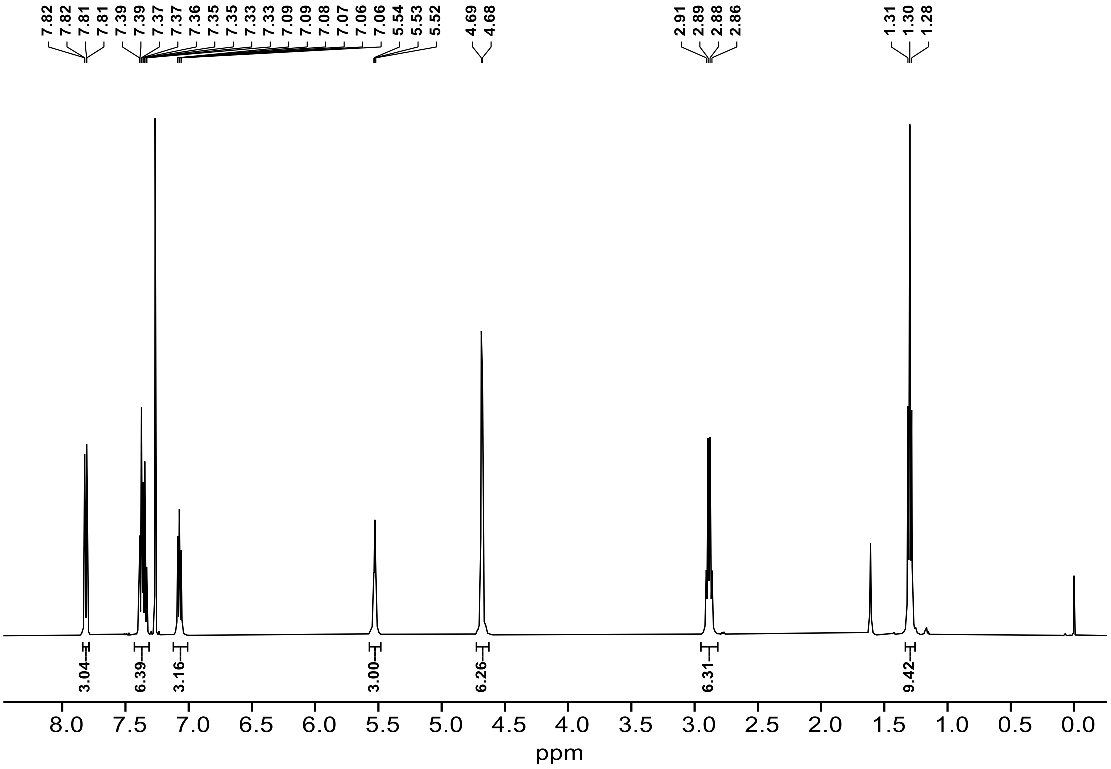

Figure S8. ^{1}H NMR spectrum (500 MHz, $CDCl_3$) of compound **1**.

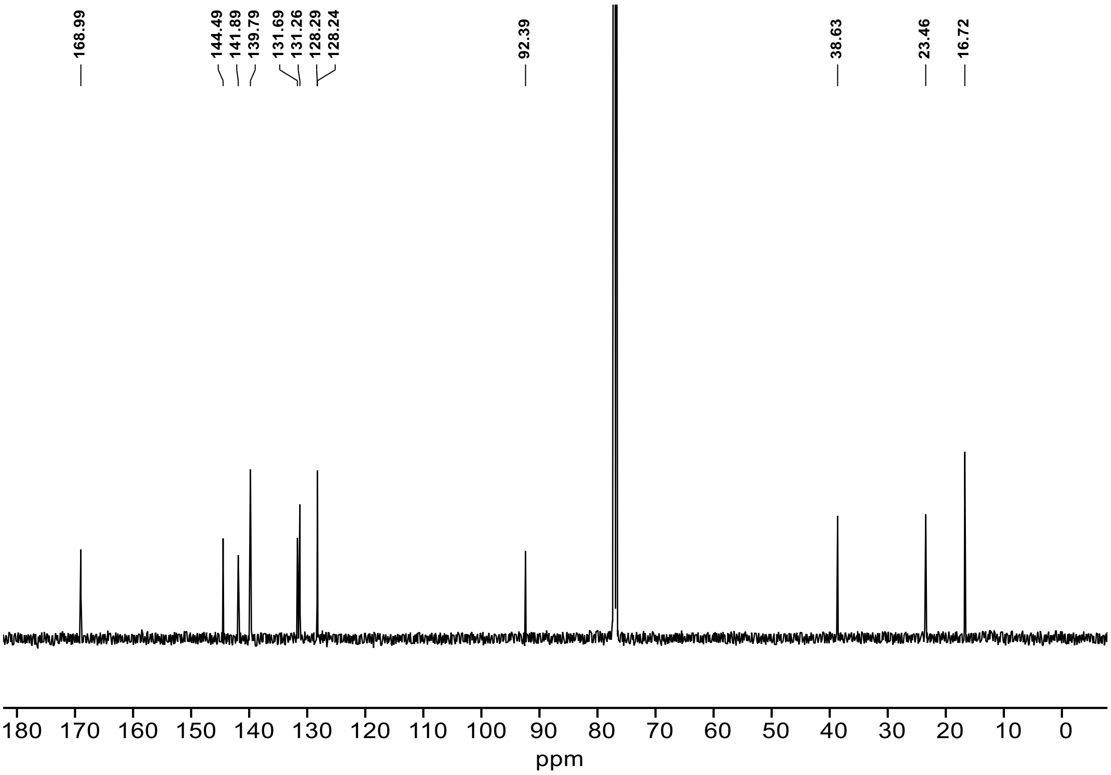

Figure S9. ^{13}C NMR spectrum (125 MHz, $CDCl_3$) of compound **1**.

6.2 ^{1}H and ^{13}C NMR spectra of compound 2

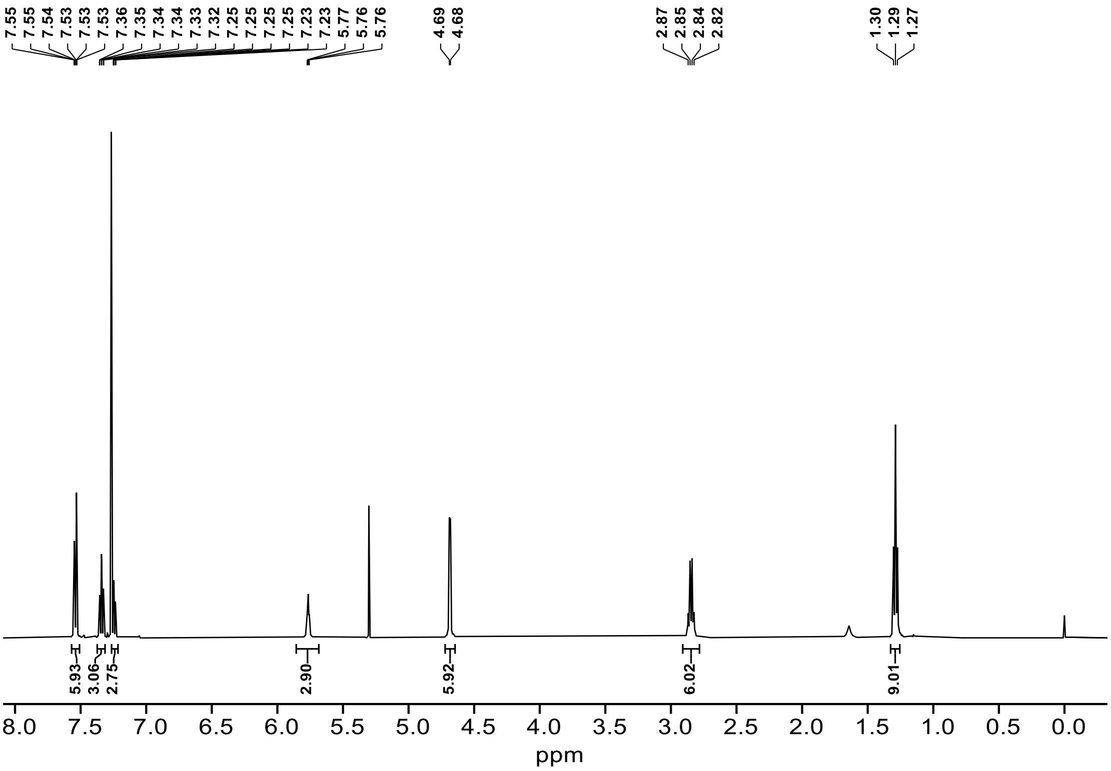

Figure S10. ^{1}H NMR spectrum (500 MHz, $CDCl_3$) of compound **2**.

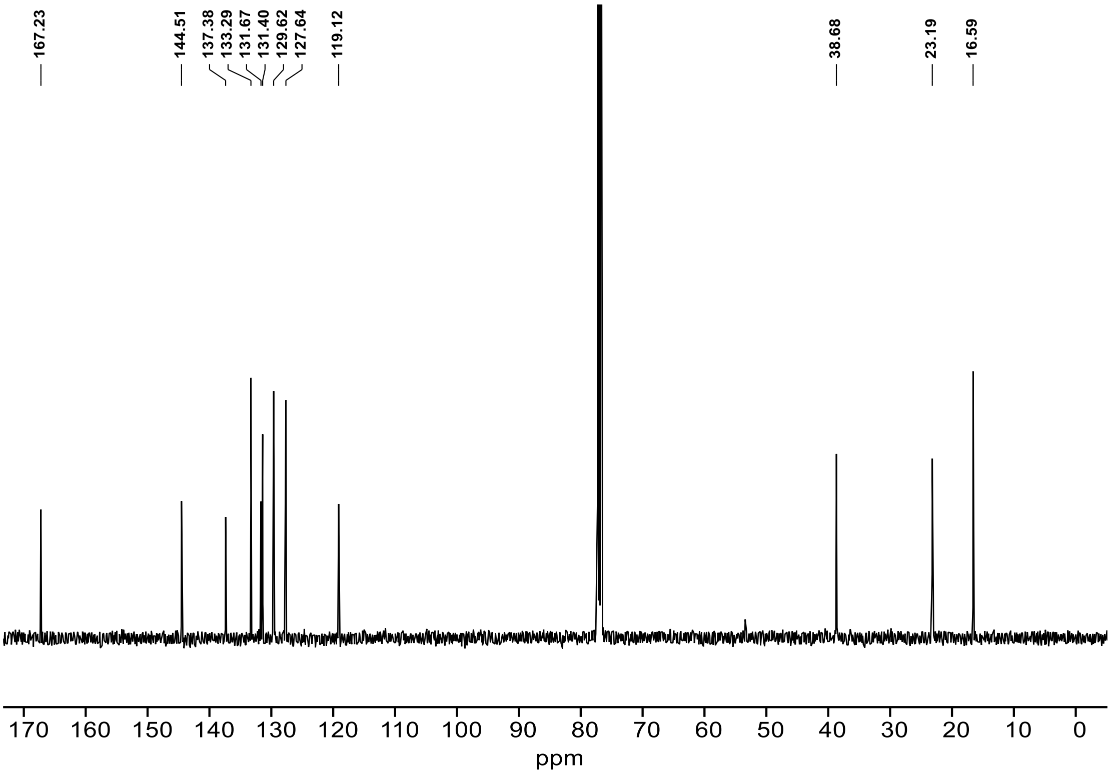

Figure S11. ^{13}C NMR spectrum (125 MHz, $CDCl_3$) of compound **2**.

6.3 ^{1}H and ^{13}C NMR spectra of compound 3

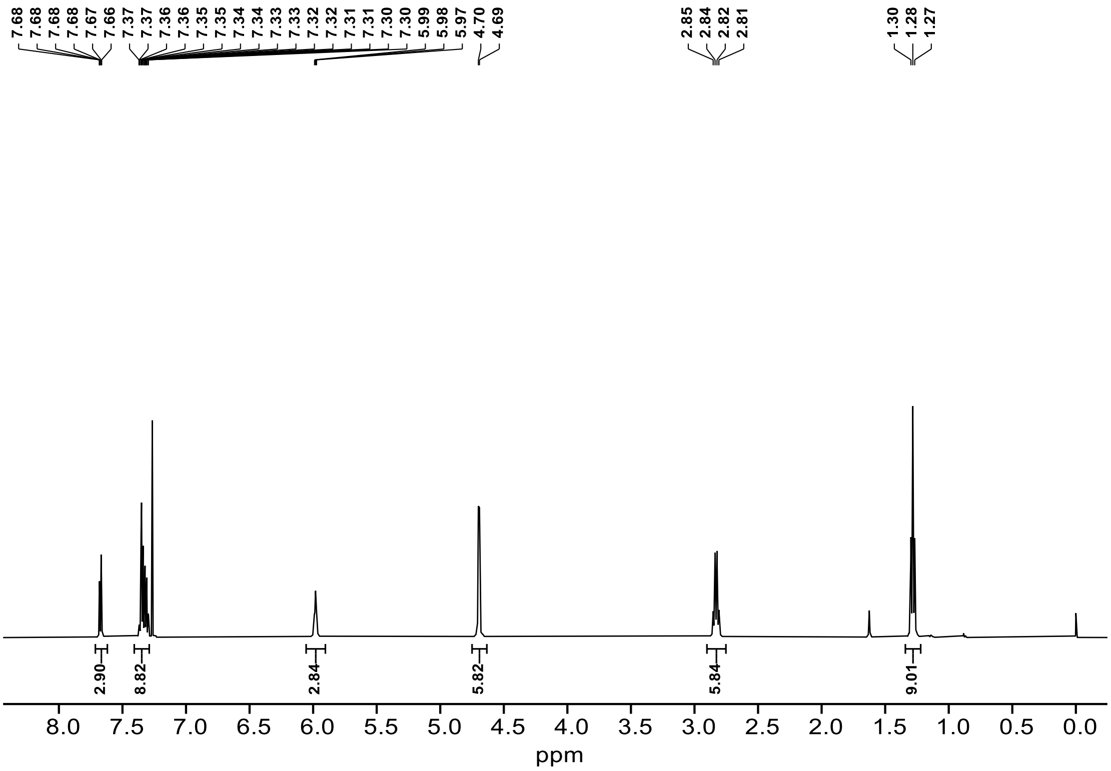

Figure S12.^{1}H NMR spectrum (500 MHz, $CDCl_3$) of compound **3**.

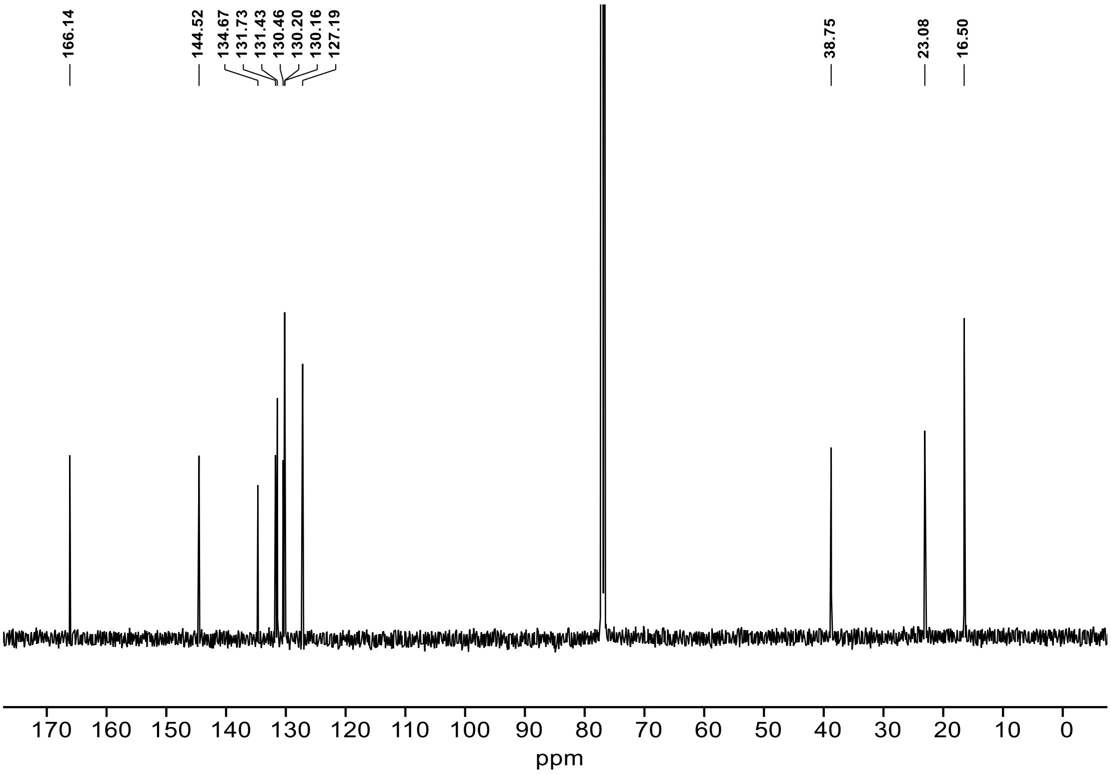

Figure S13.^{13}C NMR spectrum (125 MHz, $CDCl_3$) of compound **3**.

6.4 ^{1}H and ^{13}C NMR spectra of compound 4

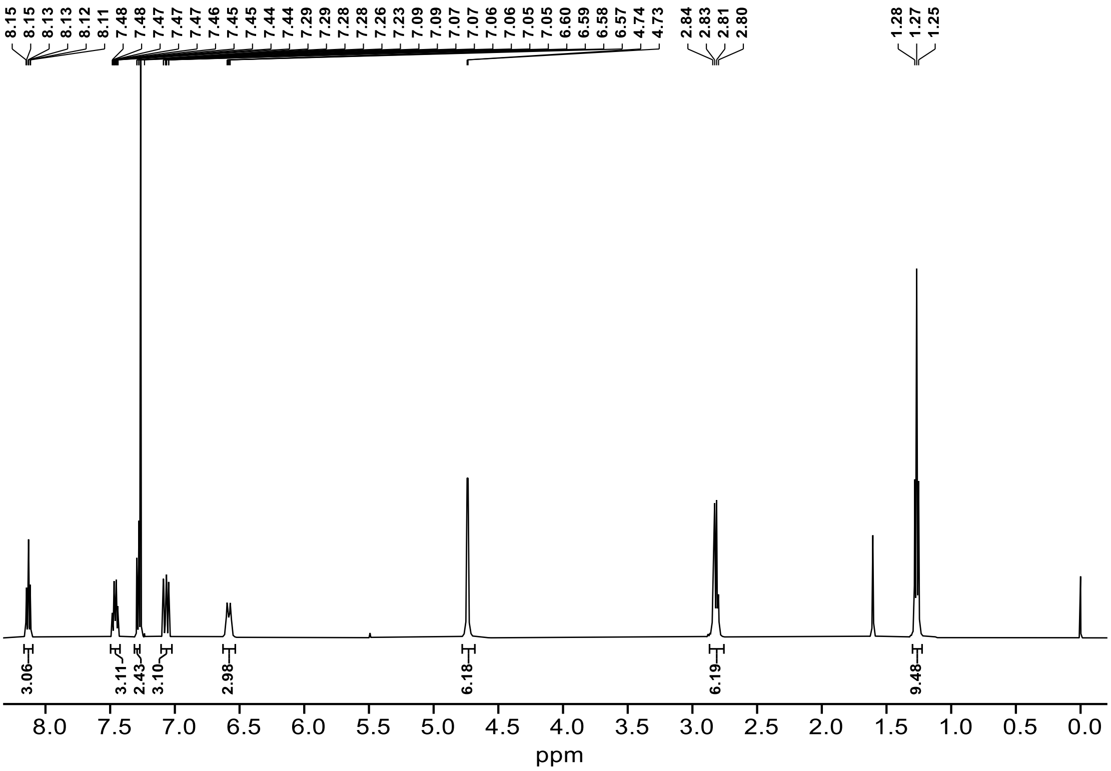

Figure S14. ^{1}H NMR spectrum (500 MHz, $CDCl_3$) of compound **4**.

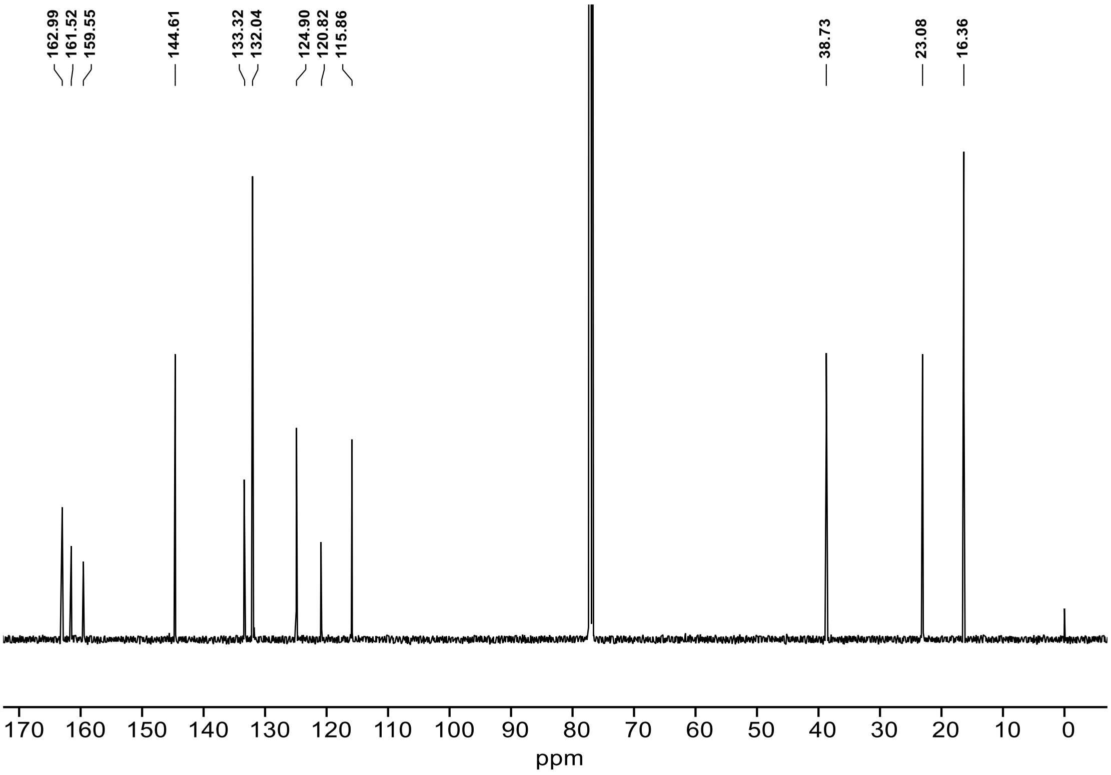

Figure S15. ^{13}C NMR spectrum (125 MHz, $CDCl_3$) of compound **4**.

6.5 ^{1}H and ^{13}C NMR spectra of compound 5

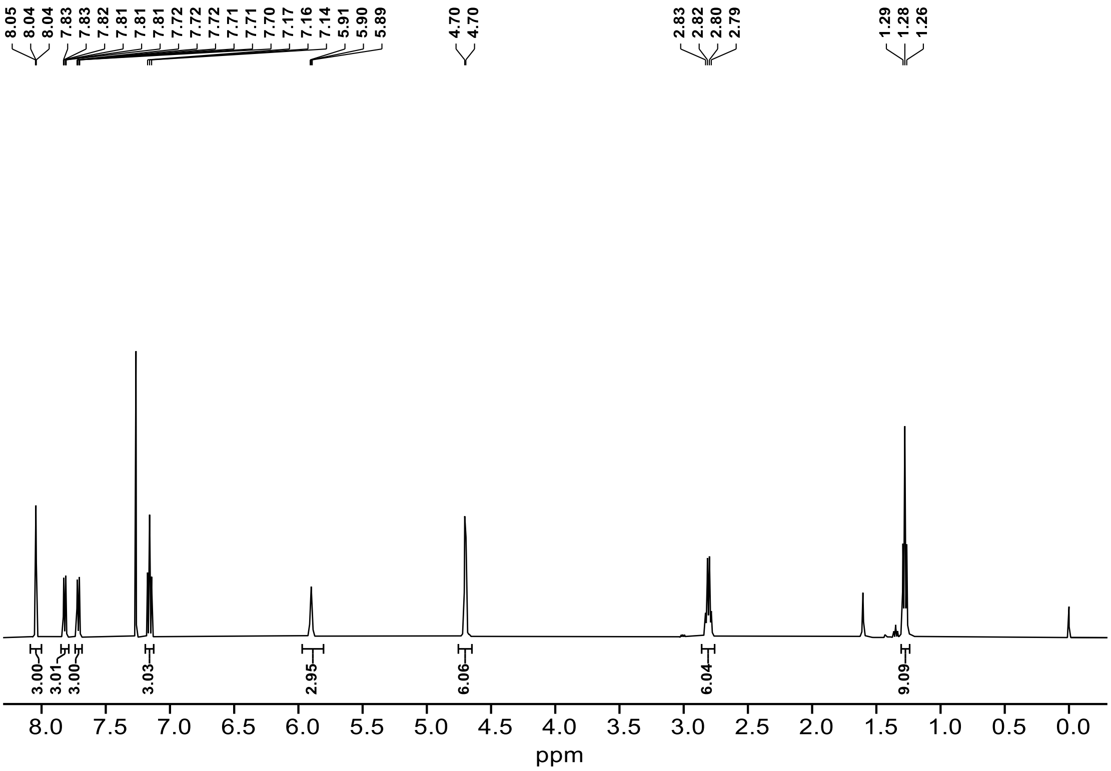

Figure S16.^{1}H NMR spectrum (500 MHz, $CDCl_3$) of compound **5**.

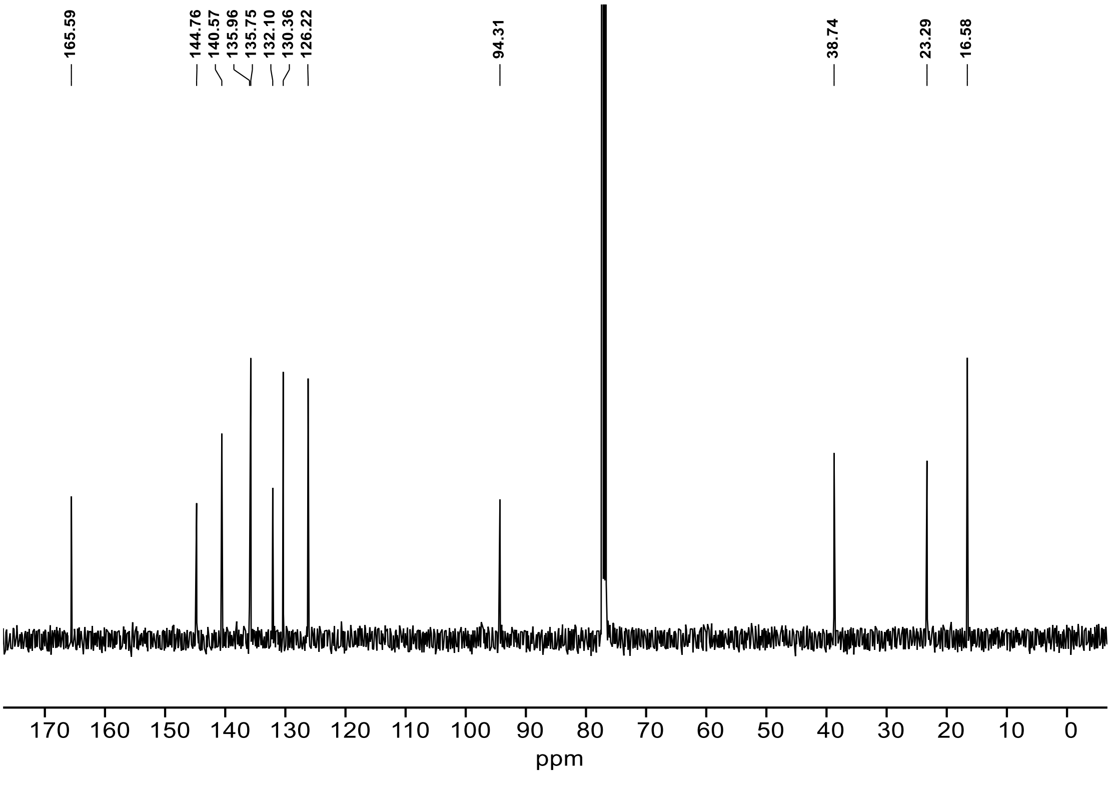

Figure S17.^{13}C NMR spectrum (125 MHz, $CDCl_3$) of compound **5**.

6.6 ^{1}H and ^{13}C NMR spectra of compound 6

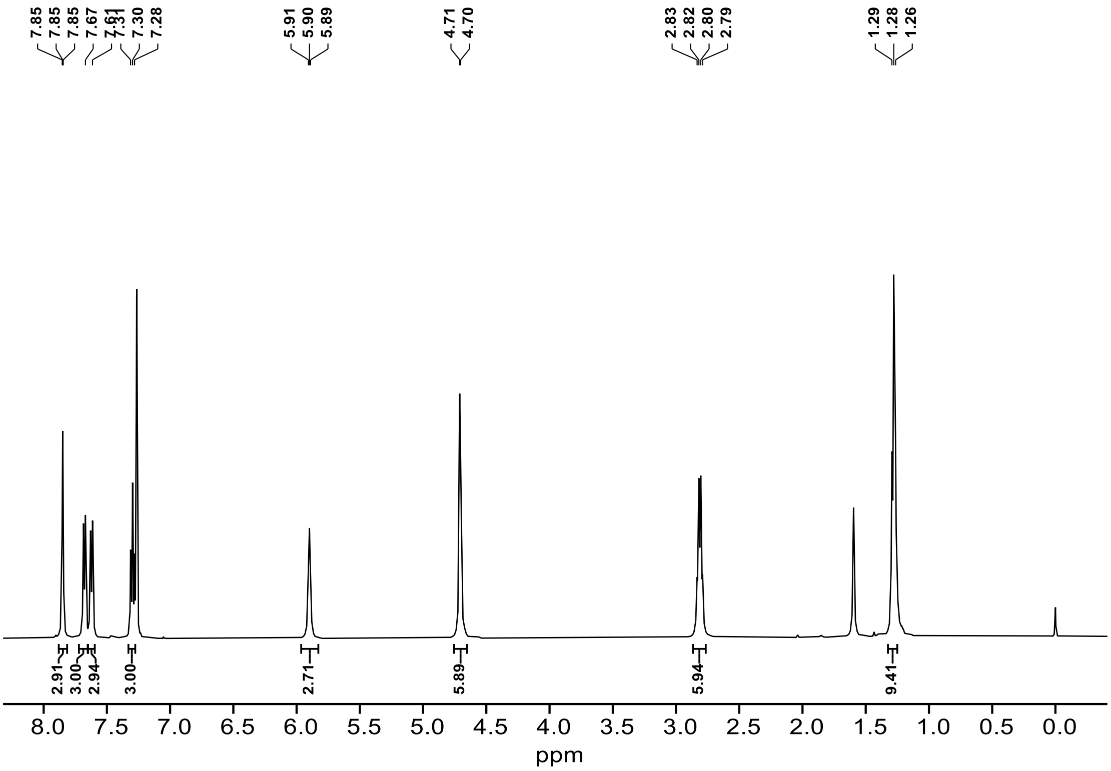

Figure S18.^{1}H NMR spectrum (500 MHz, $CDCl_3$) of compound 6.

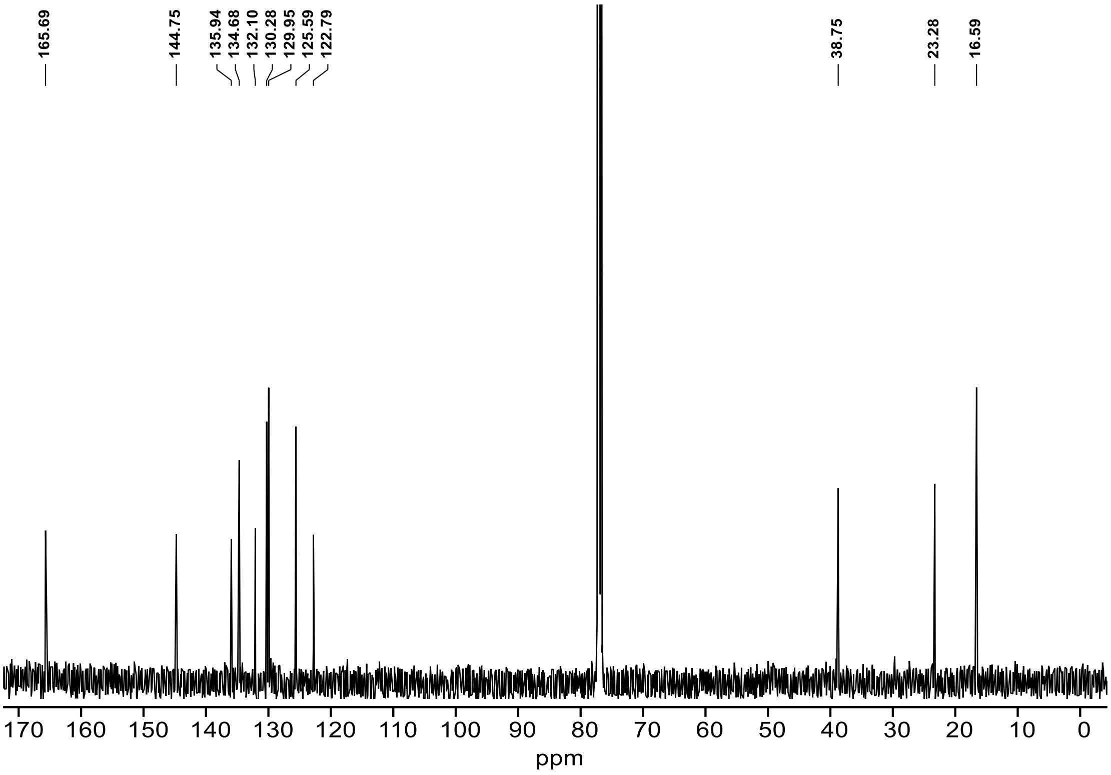

Figure S19.^{13}C NMR spectrum (125 MHz, $CDCl_3$) of compound **6**.

6.7 ^{1}H and ^{13}C NMR spectra of compound 7

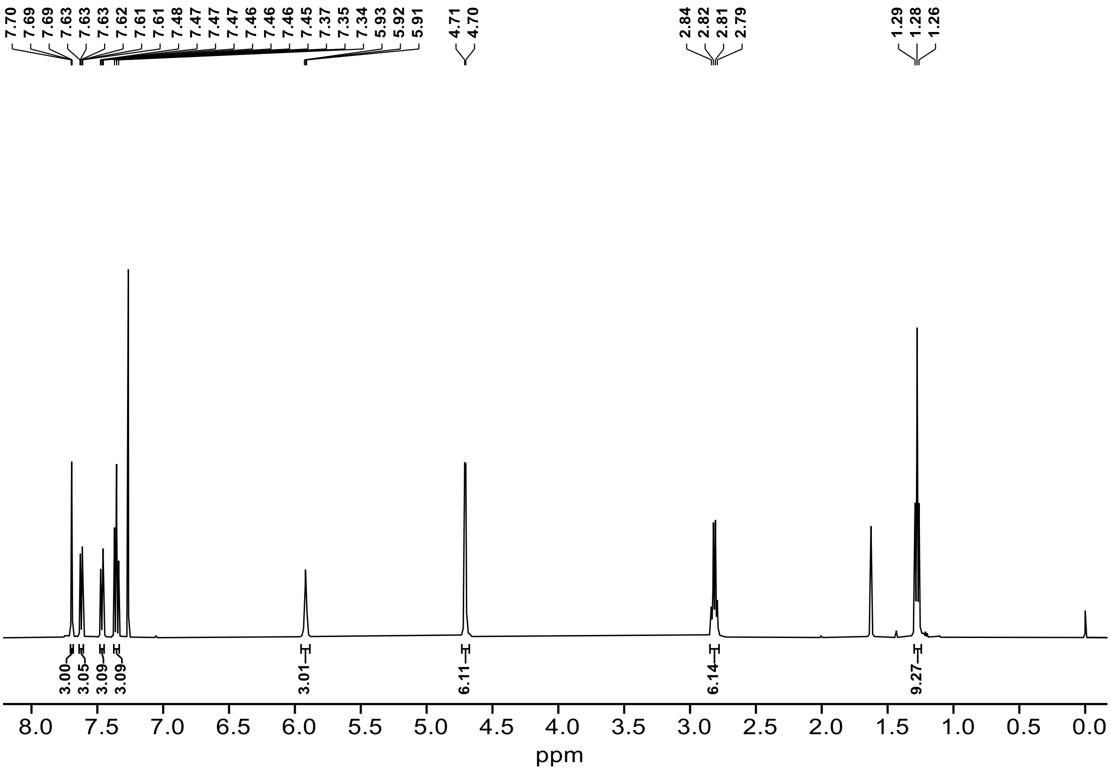

Figure S20.^{1}H NMR spectrum (500 MHz, $CDCl_3$) of compound **7**.

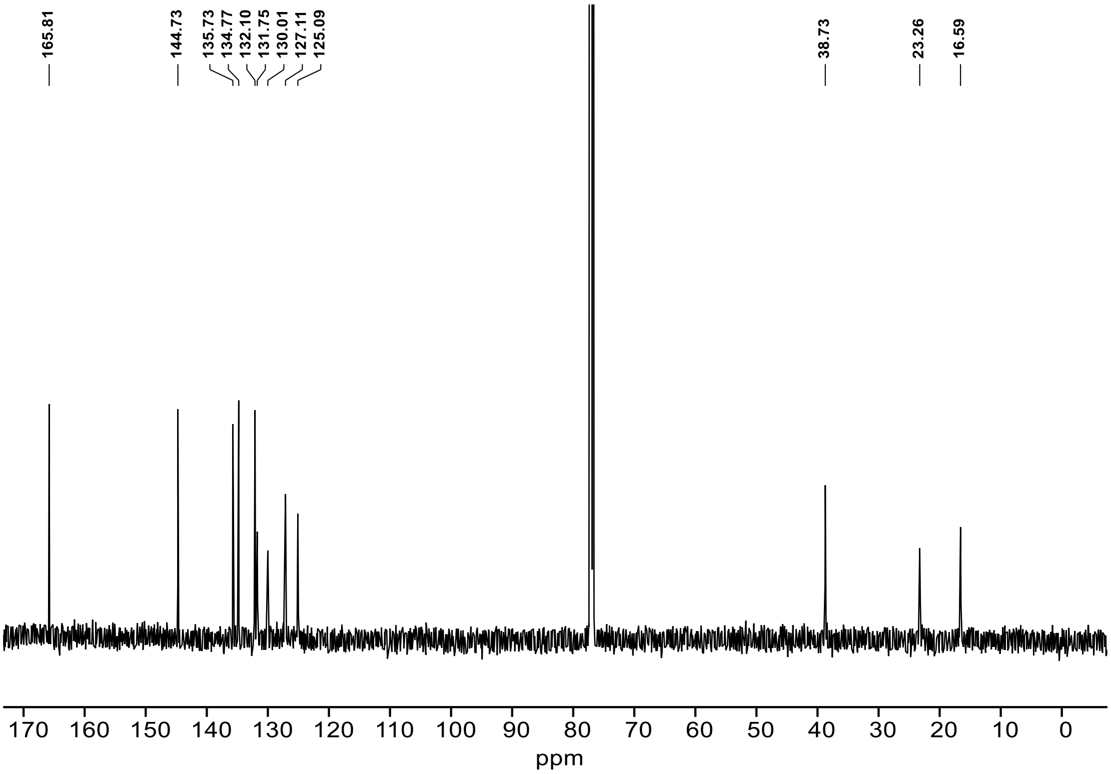

Figure 21.^{13}C NMR spectrum (125 MHz, $CDCl_3$) of compound **7**.

6.8 ^{1}H and ^{13}C NMR spectra of compound 8

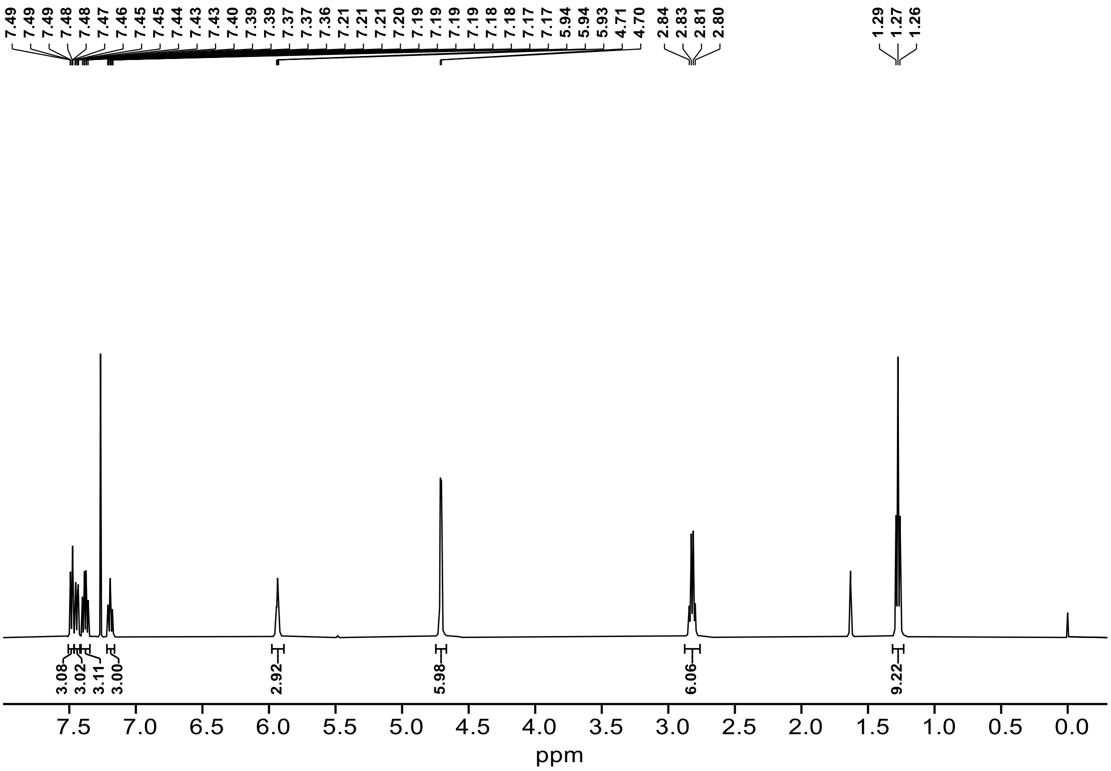

Figure 22. ^{1}H NMR spectrum (500 MHz, $CDCl_3$) of compound **8**.

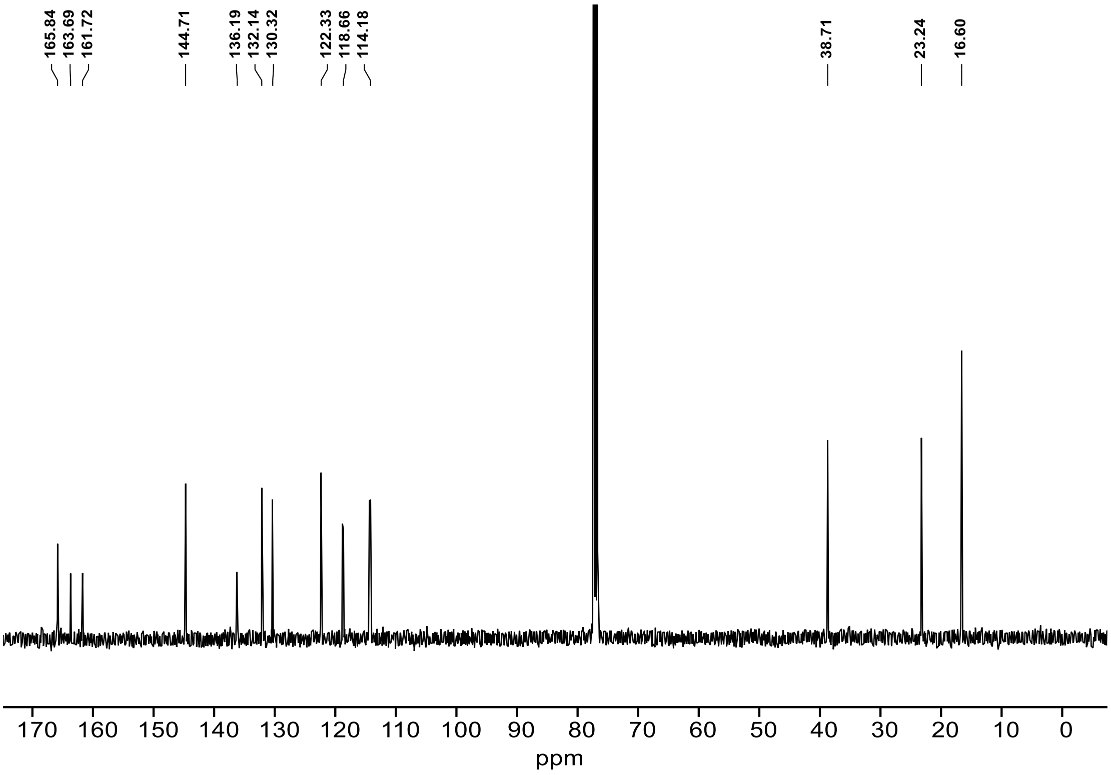

Figure 23. ^{13}C NMR spectrum (125 MHz, $CDCl_3$) of compound **8**.

6.9 ^{1}H and ^{13}C NMR spectra of compound 9

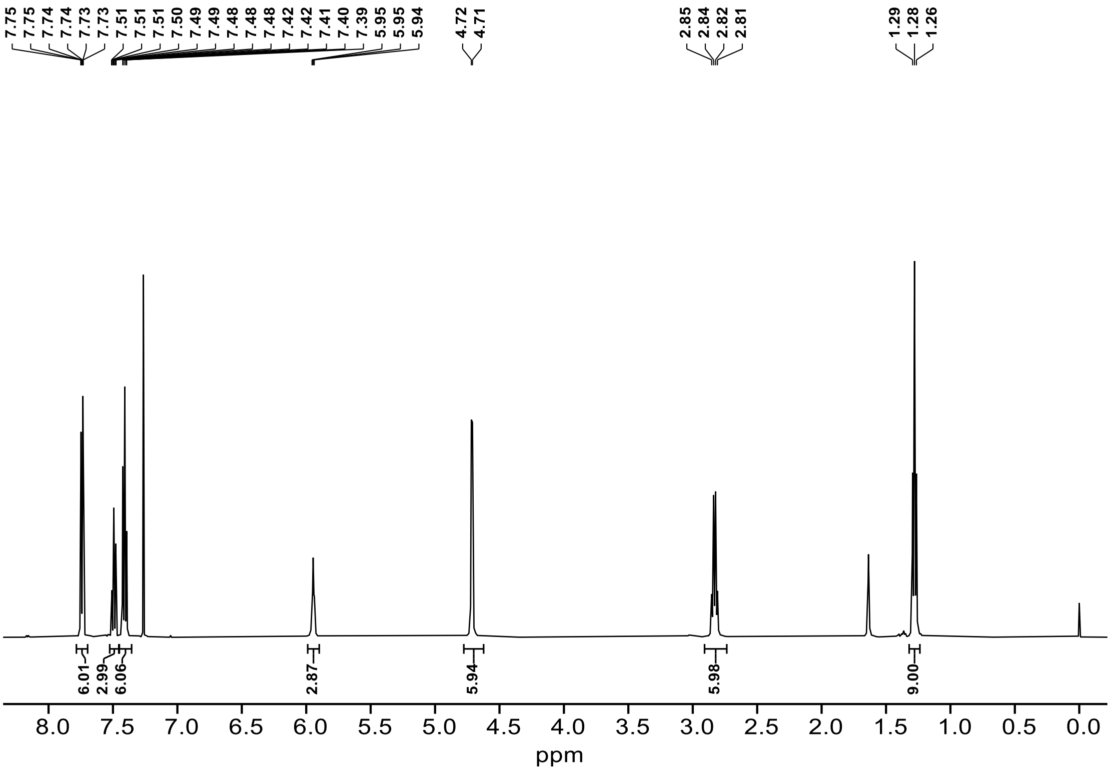

Figure 24. ^{1}H NMR spectrum (500 MHz, $CDCl_3$) of compound **9**.

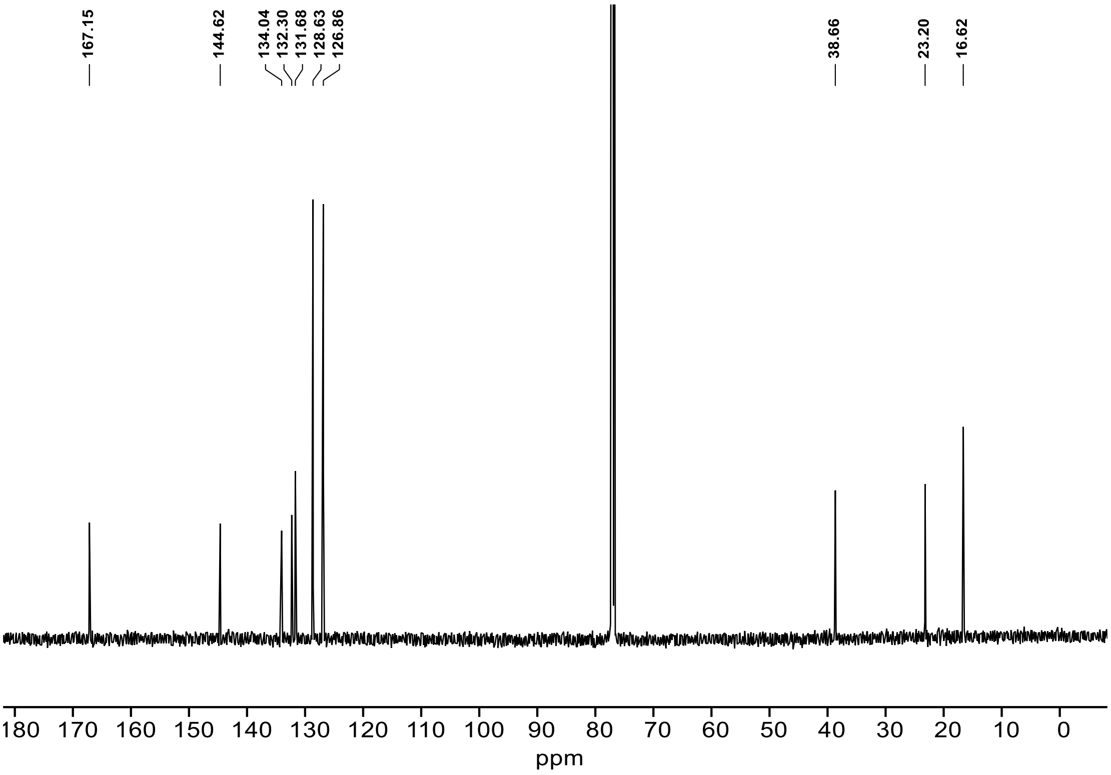

Figure 25. ^{13}C NMR spectrum (125 MHz, $CDCl_3$) of compound **9**.
